# A systematic review of the evidence on peer education programmes for promoting the sexual and reproductive health of young people in India

**DOI:** 10.1080/26410397.2020.1741494

**Published:** 2020-05-06

**Authors:** Mariam Siddiqui, Ishu Kataria, Katherine Watson, Venkatraman Chandra-Mouli

**Affiliations:** a Senior Public Health Researcher, RTI International, India, New Delhi, India; b Independent Consultant in Sexual Reproductive Health & Rights (SRHR), Singapore; c Scientist, Department of Sexual and Reproductive Health and Research, World Health Organization, Geneva, Switzerland; * Joint first authors

**Keywords:** peer education, sexual and reproductive health, young people, India, systematic review

## Abstract

In the context of a growing adolescent population globally, it is imperative to understand which interventions will most effectively advance their sexual and reproductive health (SRH). In India and globally, peer education is often utilised as an intervention for promoting the SRH of young people. Globally, the evidence of its effectiveness is mixed. A systematic review of the literature from the Indian context gave insight into the knowledge, attitudinal, and behavioural (KAB) outcomes affected by peer education, as well as the inputs, coverage, content, and context of such interventions. Out of the over 1500 publications initially identified through the database and bibliographic searches, 13 were included in the review; no quality assessment was done, given the dearth of publications matching the inclusion criteria. Analysis of the included publications highlights the multiple ways that peer education is implemented in the Indian context, as part of multi-component programmes and as a stand-alone intervention. The KAB outcomes from these initiatives are mixed, with some multi-component and some stand-alone initiatives affecting statistically significant outcomes and others not–a finding consistent with global literature reviewed for this paper. Despite the mixed results and the limited effects of behaviour relative to knowledge, this paper proposes that peer education has a place in an overall response to improving the SRH of young people. It calls for better research on peer education in India, and for research in relation to the optimal conditions for peer education to succeed in affecting KAB and other outcomes.

## Résumé

Avec une population adolescente croissante dans le monde, il est impératif de comprendre quelles interventions feront le plus efficacement progresser la santé sexuelle et reproductive (SSR) de ce groupe. En Inde et dans le monde, l’éducation par les pairs est souvent utilisée comme une intervention pour promouvoir la SSR des jeunes. Dans le monde, les données sur son efficacité ne sont guère concluantes. Un examen systématique des publications issues du contexte indien a donné un éclairage sur les connaissances, les attitudes et les pratiques (CAP) influencées par l’éducation par les pairs, ainsi que les contributions, la couverture, le contenu et le contexte de ces interventions. Sur les plus de 1500 publications initialement identifiées par des recherches bibliographiques et dans la base de données, 13 ont été incluses dans l’examen; aucune évaluation de la qualité n’a été faite, compte tenu de la rareté des publications réunissant les critères d’inclusion. L’analyse des publications incluses souligne les manières multiples dont l’éducation par les pairs est mise en œuvre dans le contexte indien, dans le cadre de programmes à multiples composantes ou comme intervention autonome. Les résultats de ces initiatives en matière de CAP sont disparates, certaines initiatives autonomes ou à multiples composantes obtenant des résultats statistiquement significatifs alors que d’autres non, une observation qui cadre avec les publications mondiales examinées pour cet article. En dépit des résultats nuancés et des effets limités des pratiques relatives aux connaissances, l’article suggère que l’éducation par les pairs a sa place dans une réponse globale pour améliorer la SSR des jeunes. Il demande des recherches de meilleure qualité sur l’éducation par les pairs en Inde et des recherches en rapport avec les conditions optimales pour que l’éducation par les pairs parvienne à influer sur les CAP et d’autres résultats.

## Resumen

En el contexto de la creciente población de adolescentes a nivel mundial, es imperativo entender qué intervenciones promoverán su salud sexual y reproductiva (SSR) de la manera más eficaz. En India y en el resto del mundo, la educación de pares a menudo se utiliza como intervención para promover la SSR de jóvenes. Mundialmente, la evidencia de su eficacia es mixta. Una revisión sistemática de la literatura del contexto indio permitió ver los resultados relacionados con los conocimientos, actitudes y comportamientos (KAB, por sus siglas en inglés) afectados por la educación de pares, así como los aportes, cobertura, contenido y contexto de las intervenciones. De más de 1500 publicaciones identificadas inicialmente por medio de búsquedas en bases de datos y en bibliografías, trece fueron incluidas en la revisión; no se realizó una evaluación de la calidad, debido a la escasez de publicaciones que reúnen los criterios de inclusión. El análisis de las publicaciones incluidas destaca las diversas maneras en que la educación de pares es aplicada en el contexto indio, como parte de programas de múltiples componentes y como intervención independiente. Los resultados KAB de estas iniciativas son mixtos, ya que algunas iniciativas con múltiples componentes y otras independientes producen resultados estadísticamente significativos mientras que otras no. Este hallazgo concuerda con la literatura mundial revisada para este artículo. A pesar de resultados mixtos, y de los efectos limitados del comportamiento con relación al conocimiento, este artículo propone que la educación de pares tiene lugar en la respuesta general para mejorar la SSR de las personas jóvenes. Insta a realizar mejores investigaciones sobre la educación de pares en India, y a velar por que las investigaciones relacionadas con las condiciones óptimas para la educación de pares logren afectar KAB y otros resultados.

## Introduction

Young people’s sexual and reproductive health (SRH) is recognised as a crucial component for progress toward global development outcomes related to education, poverty reduction and gender equality, amongst others.^1,2^ Given that young people constitute nearly one-fourth of the world’s total population, a focus on this age group is both imperative and inevitable, particularly for India, the country with the largest share of adolescents in the world.^3^

Peer education is not a new programmatic intervention for SRH. The simplicity and commonsensical nature of its rationale–that young people can more easily reach their peers with education and can discuss sensitive issues with them more easily than adults can–may be behind its prolific use in SRH programming. To place this India-focused systematic review in the context of the wider literature, we appraised the global literature, identifying reviews on the effectiveness of interventions aimed at preventing SRH problems and the behaviours amongst adolescents and young people that contribute to them. Despite the use of peer education globally, the evidence of its contribution to bringing about changes in SRH knowledge, attitudes, and behaviours is mixed.^4–37^ Further, little to no evidence exists on the possibility of peer education impacting upon other desirable changes, such as the creation of safe spaces, friendship networks, and youth empowerment. Finally, there is limited evidence on the optimal conditions for peer education programmes to be effective. This systematic review thus addresses part of the evidence gap by seeking to understand the inputs, processes, outputs, and outcomes of youth peer education interventions undertaken in the Indian context and to gain insights on their effectiveness on the above-mentioned outcomes. Specifically, the review sought to answer the changes that these initiatives have affected in relation to SRH knowledge, attitudes, and behaviours of young people. Besides the primary research question, we also addressed additional subquestions:

1. What was the content of these initiatives?
2. How were these initiatives delivered (by whom, where, with what support tools)?
3. In what context were they delivered, i.e. were they part of a wider intervention package?
4. What was the coverage of these initiatives?
5. What was the quality of these initiatives?
6. What other, if any, changes have resulted, e.g. in perception of stronger trust (attitude) and behaviour (help-seeking) from friends?

## Methods

### Search strategy

Following standard systematic review methodology, the research team developed a protocol to comprehensively collect and analyse evidence related to the research questions.^38^ As a first step, an expert advisory group was convened to help define key terms for the research (Box 1).^39–41^ Members of the group included individuals with experience in research and programme implementation on young people’s SRH in India and those with existing relationships with SRH non-governmental organisations (NGOs).

Box 1. **Key definitions**

**Sexual health** is a state of physical, emotional, mental and social well-being in relation to sexuality; it is not merely the absence of disease, dysfunction or infirmity.

Sexual health requires a positive and respectful approach to sexuality and sexual relationships, as well as the possibility of having pleasurable and safe sexual experiences, free of coercion, discrimination and violence. For sexual health to be attained and maintained, the sexual rights of all persons must be respected, protected and fulfilled.^39^

**Reproductive health** is a state of complete physical, mental and social well-being and not merely the absence of disease or infirmity, in all matters relating to the reproductive system and to its functions and processes. Reproductive health therefore implies that people are able to have a satisfying and safe sex life and that they have the capability to reproduce and the freedom to decide if, when and how often to do so. Implicit in this last condition are the rights of men and women to be informed and to have access to safe, effective, affordable and acceptable methods of family planning of their choice, as well as other methods of their choice for regulation of fertility which are not against the law, and the right of access to appropriate health-care services that will enable women to go safely through pregnancy and childbirth and provide couples with the best chance of having a healthy infant. In line with the above definition of reproductive health, reproductive health care is defined as the constellation of methods, techniques and services that contribute to reproductive health and wellbeing by preventing and solving reproductive health problems. It also includes sexual health, the purpose of which is the enhancement of life and personal relations, and not merely counselling and care related to reproduction and sexually transmitted diseases.^39^

Bearing in mind the above definition, **reproductive rights** embrace certain human rights that are already recognised in national laws, international human rights documents and other consensus documents.

These rights rest on the recognition of the basic right of all couples and individuals to decide freely and responsibly the number, spacing and timing of their children and to have the information and means to do so, and the right to attain the highest standard of sexual and reproductive health. It also includes their right to make decisions concerning reproduction free of discrimination, coercion and violence, as expressed in human rights documents.^40^

**Young people** are defined as encompassing individuals aged 10–24 years.^39, 40^ This age grouping encompasses both adolescents (10–19 years of age) and youth (15–24 years of age).

**Peer education** is a strategy whereby individuals from a target group provide information, training, or resources to their peers. These groups can be determined by social or demographic characteristics (e.g. age, education, type of work) or by risk-taking behaviour (e.g. injection drug use, commercial sex work). Peer networks can increase the credibility and effectiveness of the message being presented as they convey information to often hard-to-reach populations. Peer education is widely used and is generally a low-cost intervention. It is a good approach for conveying information in natural settings where target groups are located (e.g. schools, work sites, social gathering places such as parks or clubs), when group members are unlikely to receive services without such an approach, or when a peer is much more likely to appear credible than a non-group member (e.g. among stigmatised groups).^41^

The search strategy aimed to identify studies and evaluations of initiatives in India that included a component of SRH peer education for young people. Relevant peer-reviewed articles and grey literature (hereinafter referred to collectively as “publications”) were identified using three methods: PubMed and POPLINE database searches; bibliographic reviews; and targeted outreach to organisations. PubMed searches were conducted using the medical subject heading (MeSH) terms “sexual and reproductive health and rights”, “peer education program”, and “young people”. The POPLINE search was done using the main topics and subtopics of this review, along with the key search terms (Box 2). The bibliographies of select articles were then reviewed to identify further publications. The list of relevant organisations to target with outreach was developed in consultation with the expert advisory group and included both youth- and adult-led national and international NGOs and donor agencies.

### Inclusion criteria

The research team included publications only if they adhered to all of the following criteria: were studies and evaluations of interventions that took place in India; were published on or after 1 January 2000 and on or before 31 December 2016; the research was initiated on or after 1 January 2000; the intervention included a stand-alone or integrated peer education component focusing on the promotion of young people’s SRH; the target group was young people aged 10–24; measurements on changes in knowledge, attitudes, and/or behaviours were reported; were published in English.

Box 2. **Literature search strategy**

**PubMed** searched October 24, 2016: 1302 results

Filters applied: article type (clinical trials, evaluation studies, meta-analysis, review, systematic review), publication dates (2000-2015), species (humans), Languages (English), Ages (child: 6–12 years, adolescent: 13–18 years, young adult: 19–24 years)

(((“Sexual health” OR “reproductive health” OR “reproductive choice” OR “sexual behavior” OR “HIV/AIDS” OR “HIV” OR “acquired immunodeficiency syndrome” OR “sexually transmitted infections” OR “maternal health” OR “pregnancy” OR “abortion” OR “contraceptive choice” OR “contraception behavior” OR “family planning” OR “contraception” OR “child marriage” OR “sexual violence” OR “menstrual hygiene” OR “sexuality” OR “gender” OR “gender norm” OR “gender based violence” OR “sexual rights” OR “reproductive rights”)) AND (“peer education” OR “peer group” OR “peer mentored” OR “youth led” OR “youth run” OR “youth informed” OR “participation” OR “involvement” OR “meaningful participation” OR “programs” OR “social participation” OR “leadership” OR “community engagement” OR “process evaluation” OR “health services” OR “behavior” OR “life skills education” OR “comprehensive sexuality education”)) AND (“Adolescent” OR “adolescence” OR “youth” OR “young adult” OR “young adolescent” OR “young”)

**POPLINE** searched October 25, 2016: 205 results

Filters applied: India, 2000–2015

Main category: Adolescent Reproductive Health

- Early and unintended pregnancy
- Family Planning Programs and Services
  – Community-based Non-formal Education Programs
  – Increasing Adolescents’ Participation in Programs
  – Increasing Adolescents’ Access to Health Services
  – Unmet Need for Family Planning Services
  – Youth Friendly Clinic Services
- HIV and STIs in Adolescents
- Improving Knowledge, Attitudes, and Skills of Adolescents
  – Advocacy Campaigns
  – Life-Skills Education
  – Meeting the Needs of Married Adolescents
  – Peer Education
  – Youth Clubs/Organisations
- Sexual Behaviour

### Exclusion criteria

The research team excluded publications that were: secondary analyses of existing data sets for the purpose of presenting integrative outcomes from different research studies or programmes; discussions of literature included in contributions to theory building or critique; summaries of the literature for the purpose of information or commentary; editorial discussions that argue the case for a field of research or course of actions

### Data analysis

**Figure 1.**
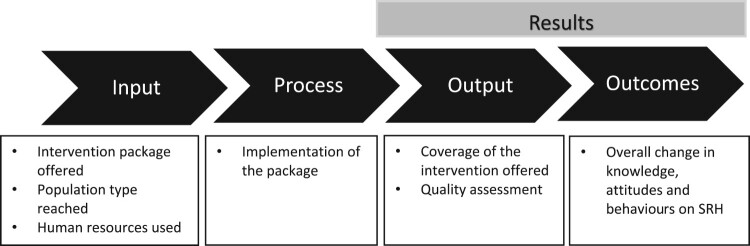
Logic model

In order to facilitate the data analysis, the research team developed a logic model (Figure 1) which, in turn, formed the basis for the review tables (Appendix A). We used the standard Preferred Reporting Items for Systematic Reviews and Meta-Analyses (PRISMA) flow diagram for reporting findings.^38^ The tabled information includes: geographical location of the study or evaluation; year of completion; name of implementing organisation; objectives of the study or evaluation; implementation (intervention package, target group, and human resources); results (outputs and outcomes); and limitations. Data entered in the review tables (Appendix A) were discussed with all authors to reach a consensus on characteristics and main findings of each publications.

Consistencies and divergences of findings across publications, methodological limitations of existing research, and knowledge and evidence gaps were identified as part of the review and synthesis. However, given the dearth of publications that fit the inclusion criteria and were accessible to the research team, the decision was made not to assess and exclude publications on the basis of quality, as it would have further limited the evidence base. The preliminary findings of the review were discussed and refined in consultation with the expert advisory group.

## Findings

**Figure 2.**
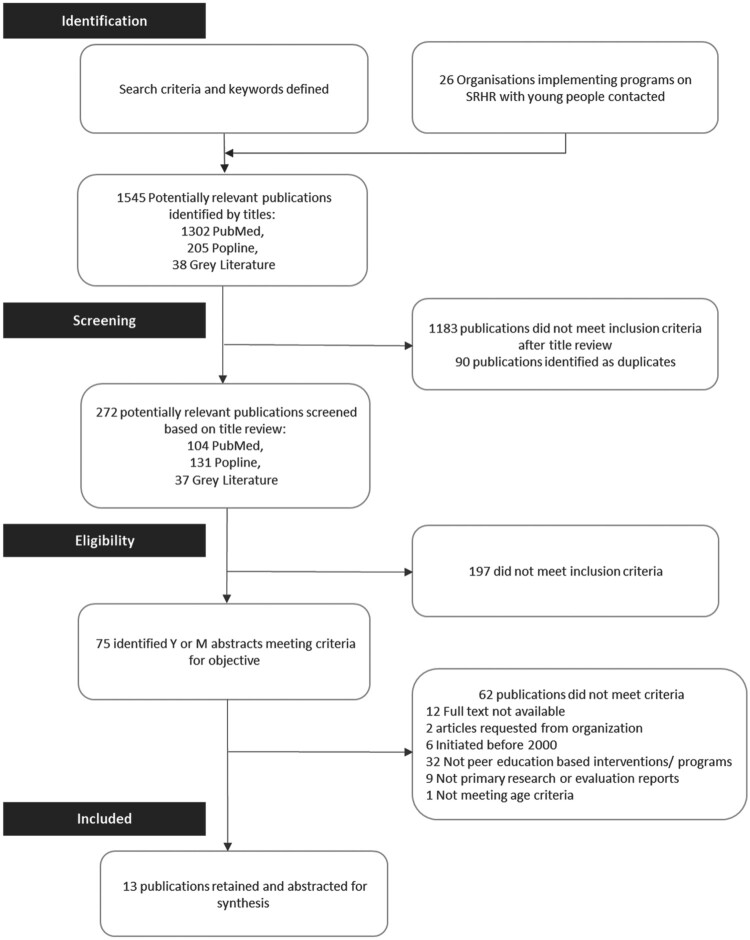
Literature search process and results

Using the above-mentioned search strategy, the team identified 1545 publications, including 38 from the grey literature (Figure 2). As a first step, the titles of all publications were screened by two authors, resulting in the exclusion of 1183 that did not meet the inclusion criteria; another 90 duplicates were also removed. As a second step, the abstracts of the remaining 272 publications were screened by the same two authors and categorised into three groups: (1) those that met the inclusion criteria (Y); (2) those for which it was unclear whether or not they met the inclusion criteria (M); and (3) those that did not meet the inclusion criteria (N). Any difference of opinion between the authors was resolved through discussion and consultation. As a third step, the 75 falling into categories Y and M were retrieved and reviewed in full by the two authors, as a result of which 62 were excluded for the following reasons: full text not available (*n* = 12) or not provided by the concerned organisation (*n* = 2); research initiated before the year 2000 (*n* = 6); no peer education based intervention or programme (*n* = 32); not research studies or evaluation reports (*n* = 9); or not meeting the age criteria (*n* = 1). Of the 13 publications in the final list, 9 are peer-reviewed journal papers and 4 are evaluation reports from the grey literature (Table 1). As our inclusion criteria include interventions initiated in the year 2000, it is likely that the publications included in this final list are skewed away from the first few years of the review period. Characteristics and main findings of these publications (labelled with letters A-M)^42–54^ are found in Appendix A.

**Table 1.** Literature search results

A^42^	Balaji M, Andrews T, Andrew G, Patel V. The acceptability, feasibility, and effectiveness of a population-based intervention to promote youth health: an exploratory study in Goa, India. J Adolesc Health. 2011;48(5):453-460.
B^43^	Parwej S, Kumar R, Walia I, Aggarwal AK. Reproductive health education intervention trial. Indian J Pediatr. 2005;72(4):287-291.
C^44^	Sebastian MP, Grant M, Mensch B. Integrating adolescent livelihood activities within a reproductive health programme for urban slum dwellers in India. 2005.
D^45^	YP Foundation. Advancing Leadership and Life Skills to Enable Young People’s Access to Sexual and Reproductive Health Information and Services. Programme Closure Report (September 2014 - January 2016). 2016.
E^46^	Child In Need Institute (CINI). Making a Difference 2012-15: Advancing Young People's Reproductive and Sexual Health and Rights though a Government-Civil Society Partnership in Four Districts of West Bengal, India. CINI;2015.
F^47^	Child In Need Institute (CINI). Report on the Impact Assessment of the Project Strengthening Rashtriya Kishore Swasthya Karyakram through Government Civil Society Partnership in West Bengal. 2016.
G^48^	Mensch B. S., Grant M. J., Sebastian M. P., Hewett P. C., Huntington D. The Effect of a Livelihoods Intervention in an Urban Slum in India: Do Vocational Counseling and Training Alter the Attitudes and Behavior of Adolescent Girls? : Population Council;2004.
H^49^	Centre for Development and Population Activities (CEDPA). SABLA – Improving well being of adolescent girls to improve their health, economic and education status. Achievements and Challenges in the implementation of SABLA scheme in Jharkhand.: CEDPA India;2013.
I^50^	Jejeebhoy S. J., Gogoi A., Zavier A. J. F., et al. The effect of a gender transformative life skills education and sports coaching program on the attitudes and practices of adolescent boys and young men in Bihar. Population Council;2016.
J^51^	Chhabra R, Springer C, Rapkin B, Merchant Y. Differences among male/female adolescents participating in a School-based Teenage Education Program (STEP) focusing on HIV prevention in India. Ethn Dis. 2008;18(2 Suppl 2):S2-123-127.
K^52^	Verma R, Pulerwitz J, Mahendra VS, et al. Promoting gender equity as a strategy to reduce HIV risk and gender-based violence among young men in India. Horizons Final Report. Washington, DC: Population Council.2008.
L^53^	Administrative Staff College of India. Evaluation of SABLA Scheme: A Report Submitted to Ministry of Women and Child Development. Hyderabad: Government of India. September 2013.
M^54^	Centre for Catalyzing Change. Endline Evaluation Report on ‘Addressing the Reproductive Health needs and Rights of Married Adolescent Couples’. May 2015.

The work described in the publications (hereinafter referred to as “initiatives”) was geographically distributed across India: two initiatives each in Jharkhand, Uttar Pradesh, and West Bengal and one each in Bihar, Chandigarh, Goa, and Maharashtra. Two studies reported on initiatives in multiple (two) states–Uttar Pradesh and Delhi National Capital Region (NCR) and Maharashtra and Uttar Pradesh, respectively. One study was a pan-India evaluation. Five initiatives were published before 2008, while eight were published between 2008 and 2016.

Non-governmental organisations (NGOs) carried out 10 of the 13 initiatives. Amongst those, six (A, D-F, H, M) were carried out by indigenous NGOs, one (C) by the Indian chapter of an international NGO, two (I, K) as collaborations between international and indigenous NGOs, and one (G) as a collaboration between the international and Indian chapters of an international NGO. Two initiatives were carried out by academic institutions (B, L), whilst the remaining initiative (J) was carried out by indigenous NGOs in partnership with an academic institution.

The included initiatives fell into three categories: quasi-experimental evaluations (*n* = 8), descriptive studies (*n* = 4), and a randomised controlled trial (*n* = 1) (Supplementary Box S1). The most commonly utilised methodologies were cross-sectional and longitudinal analyses. In six of the eight quasi-experimental evaluations, baseline, and endline analyses comparing intervention and control groups were conducted (A-C, G, J, K); the remaining two utilised such comparisons without a control group (E, M). Most quasi-experimental evaluations utilised quantitative measures (A-C, E, G, J, M), although one (K) utilised mixed-methods. The descriptive studies employed quantitative (*n* = 1) (D), qualitative (*n* = 1) (H), and mixed-method (*n* = 2) approaches (F, L). Qualitative studies used in-depth interviews and focus group discussions with beneficiaries and/or key stakeholders such as frontline health workers, programme implementers, government functionaries, family members and community members.

All the initiatives had clearly defined objectives. Nine of the 13 initiatives assessed changes in knowledge, attitudes and perceptions regarding SRH (A-G, J-K), whilst five of them assessed changes in knowledge or utilisation of SRH services (D-F, L-M). Seven assessed changes in attitudes such as gender-equitable attitudes; in perceptions such as emotional health, wellbeing, and improvements in self-confidence and leadership skills; and/or in behavioural outcomes relating to condom and contraception usage, self-reported violence, and substance use (tobacco, cigarettes, or alcohol) (A, G, I, K-M).

### Implementation

Across the 13 studies, the implementation of peer education interventions varied widely. The following variables affecting implementation were considered by this review, including: (1) the SRH content of the information provided by peer educators; (2) the wider initiative within which peer education was included; (3) the modes of delivery; (4) the target population groups; and (5) the human resources used to implement the project. These variables are considered in turn below.

Firstly, whilst the exact content of the SRH information provided by peer educators was difficult to determine, the analysis indicates varying degrees of comprehensiveness. This variation in content and delivery of the initiatives and the changes in knowledge and attitudes towards SRH are not comparable. For most initiatives, it is apparent that peer educators provided general reproductive health information, though this was not defined clearly or consistently. Sexual and reproductive health topics common to many of the initiatives were: menstruation, puberty, contraception, sexually transmitted infections (STIs), including HIV, and pregnancy. Less commonly, the peer educators provided information on birth spacing, laws relating to child marriage and/or abortion, relationships, violence, sexuality, and gender. In most initiatives, peer education expanded beyond SRH to include other public health and development topics too. The most common additional topic covered was nutrition, whilst livelihoods, savings formation, substance use, sports, and adolescent health (general) were addressed by a few initiatives.

Secondly, more often than not, peer education was one amongst several interventions aimed at achieving programmatic objectives related to young people’s knowledge, attitudes, and behaviours. Out of the 13 initiatives included in this review, two reported the use of peer education as a stand-alone intervention, whilst 11 reported it as one intervention amongst two or more. Each multi-component programme used a combination of interventions in addition to peer education. Some of the additional components were directed at frontline workers, including teacher training (*n* = 1) and capacity-building for health workers (*n* = 2), whilst those directed at adolescents included: delivery of educational sessions in schools by teachers or public health nurses (*n* = 2); livelihoods and/or vocational training (*n* = 4); savings formation (*n* = 2); communication skills training (*n* = 1); community outreach and sensitisation (*n* = 3); service delivery (*n* = 1); and sports coaching (*n* = 1).

Thirdly, between the initiatives, there was great variety in the settings, materials, and delivery modes. The majority were delivered in out-of-school settings in places close to where young people live or congregate, such as community centres, *anganwadi* centres (type of rural child care centre), and homes of peer educators who work or live in slums. In two initiatives, peer educators were operating in school settings. Whilst most initiatives did not elaborate on the content or format of the materials available for peer educators, one publication mentions the use of a flip book with relevant case studies, whilst others mention training curricula or modules. In relation to delivery modes, in nine initiatives peer educators gave group informational sessions, rather than speaking to young people one-on-one, which was the modality reported in the four remaining publications.

Fourthly, whilst all the initiatives used peer education to reach young people, specific cohorts were targeted through each. The most common group targeted were adolescent girls in the 10–19 age bracket, or a subset thereof. Only two initiatives targeted young men specifically, and one targeted married couples. Most of the initiatives addressed young people younger than 20 years, although four included young people older than this and, in one case, up to 29 years of age. The target groups included a mixture of in-school and out-of-school young people, as well as urban and rural residents. Several of the studies made reference to targeting poor, low-income or slum areas.

Lastly, in addition to peer educators, in multi-component initiatives other cadres of workers were involved in delivering information or complementary interventions. Some initiatives built upon existing parts of the health, community development or education systems in India (e.g. community health workers, *anganwadi* workers, SABLA Scheme workers, teachers, child development officers) for implementation, whilst others drew upon staff members within non-governmental agencies. Implementing agencies also drew upon their own staff members, in addition to consultants, researchers, and local NGOs, for delivery.

### Coverage and quality

Coverage of these interventions was assessed based on the number of people within the target population reached by the peer educators. In 11 initiatives, peer educators reached out to young people who were a part of the intervention, whereas the remaining two initiatives had no information on coverage (C, H). Amongst the 11 initiatives that mentioned coverage, four did not specify the denominator population (D, E, J, M). The scale of these initiatives varied considerably, with the number of young people reached by peer educators ranging from 84 to 4,811,264.

None of the initiatives reported on the quality of the content or delivery of peer education. Five initiatives did conduct process monitoring (A, E, F, H, L). In one of these initiatives, on-site supervision was done along with weekly review meetings to assess intervention delivery quality (A), whereas two further initiatives developed a monitoring mechanism involving multiple stakeholders to ensure effective implementation (F, L).

### Changes in knowledge

Whilst 11 publications–including both multi-component and stand-alone peer education initiatives–reported increases in knowledge, just nine reported increases in SRH knowledge. These increases in SRH knowledge related to pubertal changes, menstrual hygiene, reproductive tract infections, STIs, and HIV; the existence of SRH services, including those for adolescents; and SRH practices such as contraceptive use (A-G, J, M). An initiative implemented by the Centre for Catalysing Change (C3) that carried out peer education for married couples in Jharkhand (M) reported significant increases in knowledge between baseline and endline surveys in relation to a variety of different topics, including knowledge of family planning methods (contraceptive pills: 20% to 91%; condoms: 39% to 97%); awareness of pregnancy detection kits (28% to 86%) and their use (20% to 56%); awareness of complications in pregnancy and childbirth often leading to maternal death as a consequence of early pregnancy (wives: 67% to 87%; husbands: 74% to 96%); and awareness of the benefits provided by the Government of India in case of institutional delivery (e.g. free transport to and from the health facility: wives 21% to 100% and husbands 36% to 97%). The *Yuva Mitr* initiative, implemented by indigenous NGO Sangath, demonstrated that young people reached by peer educators had higher levels of knowledge of and more favourable attitudes toward SRH [OR = 1.55, 95% CI: 1.06–2.28 (rural community) and OR = 1.46, 95% CI: 1.09–1.97 (urban community)] in comparison to young people reached by teachers (A).

One of the two stand-alone peer education initiatives reported change in SRH knowledge (J). This initiative assessed changes in HIV and AIDS knowledge and found that young people who received the intervention had higher knowledge scores (*p* ≤ 0.001). Additionally, young girls scored significantly higher on HIV and AIDS knowledge in comparison to young boys (*p* < 0.05).

### Changes in attitudes

Only four of the 13 initiatives measured changes in SRH attitudes or beliefs (A, J, K, M). Among these, two were stand-alone (J, K) and two were multicomponent (A, M) initiatives. The STEP initiative (J) reported that young people reached by peer educators had significant increases in positive attitudes toward consistent condom use (*p* < 0.001) and positive beliefs towards people living with HIV (*p* < 0.001). The *Yaari-Dosti* initiative, which targeted young men aged 15–29, also reported more positive attitudes towards people living with HIV (*p* < 0.05) (K). Favourable attitudes were reported by the C3 married couple initiative, which reported changes amongst both young women (46% to 93%) and their husbands (51% to 92%) in relation to attitudes toward visiting a health facility for symptoms of reproductive tract infections (M).

Overall, the two stand-alone (J, K) and one multicomponent (A) initiatives reported statistically significant positive changes in attitudes among young people who received the interventions. One other multicomponent intervention (M) reported positive changes in attitudes; however, the initiative did not provide information on whether those changes were statistically significant.

### Changes in behaviours

Seven of the 13 initiatives (A, F-I, K, M) reported changes in behavioural outcomes; however, only four of these initiatives reported changes in SRH-related behaviours—one stand-alone peer education initiative (K) and three multicomponent initiatives (A, F, M). The outcomes reported by the initiatives included increases in reporting sexual health problems or menstrual problems for young women (A, F, K); help seeking from health care providers (A, M); and visiting adolescent-friendly health services for SRH (M). The *Yaari-Dosti* initiative reported several other behavioural changes, including improvements in communication with one’s partner on condoms, sex, STIs, and/or HIV (*p* < 0.05); increased condom use (*p* < 0.05); and positive trends on the Gender-Equitable Men scale scores associated with a decrease in HIV/STI risk behaviours among the young men reached by peer educators (K). Amongst these initiatives, only the stand-alone peer education intervention reported statistically significant changes in behavioural outcomes among young people (K). None of the three multicomponent initiatives reported statistically significant changes in behaviours (A, F, M).

### Other changes

In addition to changes in SRH knowledge, attitudes, and behaviours, some initiatives reported knowledge, attitudinal, and behavioural changes in relation to other areas, including gender-based violence, child marriage, livelihood, and savings formation, nutrition, substance use, community sensitisation, and advocacy.

#### Knowledge

Six of the 13 initiatives reported changes in knowledge on non-SRH-related issues such as nutrition, substance use, emotional health, and gender equity (A, E, F, H, I, L). Four of these initiatives reported increases in knowledge about nutrition, anaemia and/or intake of iron-rich foods and folic acid tablets for young women (E, F, H, L). For example, one initiative reported young people reached by the National Adolescent Health Programme (*Rashtriya Kishor Swasthya Karyakram*) in one of the districts had increased knowledge of iron-rich foods (97% vs. 76%); were more likely to have heard of anaemia (89% vs. 48%); received information on nutrition (79% vs. 24%) and were aware of their own weight (75% vs. 62%) compared to those not reached by the national programme (F).

Three of these six initiatives reported increased awareness of the right to education, life skills education, legal age of marriage, and/or various legal and other options for women who experience marital violence (E, F, I). One initiative reported increased knowledge between baseline and endline surveys of the legal age of marriage for girls (30% to 96.5%) and boys (26% to 77%) (E). Another reported higher levels of awareness among young people on their right to education, equal rights between boys and girls and available child protection services (F).

#### Attitudes

Other than those noted above, the most common attitudinal change affected by the initiatives was in relation to gender equality; three of the 13 initiatives–including one stand-alone peer education initiative (K)–reported statistically significant changes towards egalitarian gender role attitudes and, at the same time, less support for inequitable norms (C, I, K). The *Do Kadam Barabari Ke* initiative (I), for example, reported that young men reached by peer educators expressed more egalitarian attitudes and notions of masculinity (*p* = 0.04), rejected men’s or boys’ right to exercise controlling behaviours over women or girls (*p* = 0.003), and rejected men’s or boys’ right to perpetrate violence on a woman or girl (*p* = 0.002). Moreover, the young men believed their peers would respect them for demonstrating non-traditional behaviours in at least three of the following four situations: respected by friends if a boy talks about his problems with his friends or peers; helps his mother do her housework; walks away from a fight; and refuses to physically abuse his wife even if she disobeys him (*p* = 0.04) (I).

#### Behaviours

Four of the 13 initiatives reported changes in non-SRH behaviours (A, C, G, L), all of which were multicomponent initiatives. Three of these initiatives reported increases in autonomy amongst young women, which was demonstrated by improvements in vocational skills and livelihood activities, work aspirations, physical mobility within the community and/or communication skills (C, G, L). One initiative reported a decrease in the perpetration of physical violence among young people reached by the intervention (A).

## Discussion

This paper sets out to answer a set of interrelated research questions regarding the use and effectiveness of peer education in achieving sexual and reproductive health knowledge, attitudinal, and behavioural outcomes in India. Although the quantity and quality of the evidence are limiting, this review has been able to highlight important trends and define a framework for future research.

In relation to the first three additional research questions, this review found that the way in which peer education has been utilised varies greatly in terms of content, delivery, and context in India. There is no standardised model of peer education, and the majority of initiatives combine it with other interventions such as health service delivery, sports coaching or vocational training for young people. The next two additional research questions on coverage and quality were difficult to assess given the dearth of data. Whilst numbers of young people reached through peer education were available for 11 publications–and noteworthy in several–four were missing denominators. Further, the quality of peer education was not assessed systematically in any of the initiatives.

In relation to the changes affected by the initiatives, success varied in terms of impact on SRH knowledge, attitudinal, and behavioural outcomes. In relation to SRH knowledge, nine of the 13 initiatives reported increases, with four of these reporting statistically significant results (A, B, G, J). Of the nine, eight were multi-component initiatives (A-G, M), and one was a stand-alone peer education initiative (J). Changes in SRH attitudes were reported by just four initiatives; two of these were multi-component (A, M) and two were stand-alone (J, K). The two stand-alone (J, K) initiatives and one multicomponent (A) initiative reported statistically significant attitudinal change. Lastly, SRH behavioural outcomes were reported by four initiatives (A, F, K, M), one of which was a stand-alone peer education intervention (K). Only the stand-alone peer education intervention reported statistically significant changes in behavioural outcomes amongst young people (K). Changes in non-SRH knowledge, attitudes, and behaviours were also reported in many publications, and notably so in relation to knowledge of nutrition and changes in gender-equitable attitudes. Overall, changes in the desired direction in relation to knowledge, attitudes, and behaviours occurred in some multicomponent and stand-alone interventions, but not in others.

To place the findings of our India review in perspective, we utilised our global review of reviews (Table 2). These reviews were published between 2001 and 2014^4–13^ and had been cited in two state-of-the-art reviews.^14,15^ They included published reports of research studies and evaluations carried out in high-,^16–27^ middle- and low- income countries.^28–37^ Twenty-two initiatives–10 of which were from low- and middle-income countries–provided information on the effect of peer education interventions when delivered as stand-alone interventions. Changes in knowledge, attitudes, and behaviour as a result of stand-alone peer education are mixed (Table 3). Of those initiatives reporting changes, positive results in knowledge were seen in nine of 15 initiatives (60%); in relation to attitudes, positive change was seen in six of 11 initiatives (54.5%); and in relation to behaviour, in six of the 14 initiatives (37.5%). In other words, the global review illustrates that peer education has been shown to be more effective in improving knowledge and attitudes than in promoting healthier behaviours in some initiatives, but not in others, a finding that is consistent with the India review. In the India review, whilst peer education was part of a number of multi-component initiatives in which positive changes were reported, it was also part of initiatives in which such changes were not reported. However, it is worth noting that peer-education-only initiatives from India did report statistically significant results in relation to knowledge (J), attitudes (J, K), and behaviours (J, K).

**Table 2.** Global review of reviews studies

Study/evaluation	Changes in knowledge	Changes in attitudes	Changes in behaviour
**High-income countries (n = 12)**			
Smith^16^	+	n/a	+
Fergusson^17^	+	n/a	-
Brindis^18^	n/a	n/a	/
Borgia^19^	+	n/a	-
Stephenson^20^	-	n/a	-
Kleiber^21^	-	-	-
Merakou^22^	-	+	-
Caron^23^	n/a	+	/
Fisher^24^	-	-	/
Mellanby^25^	+	+	n/a
Kegeles^26^	n/a	n/a	+
Ozer^27^	/	/	n/a
**Low- and middle-income countries (n = 10)**			
Agha^28^	+	/	/
Cartegena^29^	+	+	+
Brieger^30^	n/a	/	/
Merati^31^	n/a	+	+
Nastasi^32^	/	n/a	n/a
Speizer^33^	+	n/a	+
Mitchel^34^	n/a	n/a	n/a
Gao^35^	+	n/a	n/a
Kinsler^36^	n/a	+	+
Ozcebe^37^	+	n/a	n/a

Abbreviations: +, positive change; -, no change or no statistically significant change; /, mixed impact; n/a, not researched/reported

**Table 3.** Summary of review findings

Positive change in	mixed results	no effect	no change reported
*Knowledge*			
9	2	4	7
*Attitude*			
6	3	2	11
*Behaviour*			
6	5	5	6

Our review of Indian peer-education initiatives did not directly address the optimal conditions for the success of peer education. On the other hand, three other systematic reviews examined what it takes to ensure that peer education programs are effective—Tolli et al., Maticka-Tyndale et al. and Gottschalk et al., although only Tolli et al. did so in any depth.^7,10,13^ Further research on the optimal conditions for success for peer education is needed to provide solid evidence for designing effective programs in India and across the globe.

Another pertinent research question emerging from both India and global reviews is whether the fields of global health and human rights are measuring the “right” things in relation to peer education. To date, evaluations and research of peer education have judged its effectiveness primarily on changes in knowledge, attitudes, behaviours, and, in some cases, health outcomes. Whilst these measurements are not without value, it is important to further explore the potential of peer education to contribute to a range of desirable health and rights outcomes, including young people’s awareness of their rights to access information and services; legitimation of dialogue on previously-taboo SRH issues; young people’s awareness of where and how to seek help and their confidence in doing so; improvement in communication between peers, as well as between parents and young people; and enhancement of social networks.

## Conclusion

Peer education has been employed in India and around the world in a variety of ways to bring about changes in the SRH knowledge, attitudes and behaviours of adolescents and young people. While the published literature on this in the Indian context is uneven in quality, there are clear indications that it has contributed to improvements in these areas in some–but not all–initiatives. Now is not the moment to give up on peer education, but, rather, to better understand the role it could play within public health and human rights initiatives. Taking the next step forward in programming for adolescents and young people in India and globally will require dialogue regarding which outcomes peer education programmes could reasonably be expected to contribute to and the conditions under which they can be achieved.

## Acknowledgments

Venkatraman Chandra-Mouli conceived the paper. Suneeta Krishnan, Mariam Siddiqui and Ishu Kataria prepared the first draft. Venkatraman Chandra-Mouli provided feedback; Mariam Siddiqui and Ishu Kataria worked with him to prepare the second draft. Suneeta Krishnan dropped out of the process. Venkatraman Chandra-Mouli then worked with Mariam Siddiqui, Ishu Kataria and Katherine Watson to prepare the final draft. All authors reviewed and approved the final draft.

## Supplementary Material

Supplementary Box S1

## Appendix

**Table A1.** Characteristics and main findings of research studies and evaluation reports

**Location**: This is the state/territory in India in which the study or evaluation was conducted. **Year**: This is the year that the study or evaluation was completed. **Organisation name and type**: In this column, the name of the implementing and supporting organisations are included alongside a description of the type of organisation (e.g. indigenous NGO, international NGO, academic institution). **Objectives**: The objectives are copied directly from the studies themselves, without making changes to the wording. **Design**: This column provides information on the type of research study or evaluation used, giving an indication of the authors’ methodology. **Implementation**: This column provides information on the inputs and process of the study or evaluation. The ‘process’ cell provides a description of the content, delivery and context relevant to the study. **Results**: This column is divided into outputs (coverage, quality) and outcomes (knowledge, attitudes, practices). Coverage relates to the # of people within the target population reached. Quality relates to assessments done of the peer education (content or delivery). Where available, findings related to all 3 outcomes areas (and any additional ones) are presented with relevant statistics. **Limitations**: The limitations are taken directly from the studies and are, thus, the limitations and gaps identified by the authors themselves. Limitations relate both to the design of study and the project implementation.					
**ID (ref)**	**Location, year, organisation name and type**	**Objectives and design**	**Implementation**	**Results**	**Limitations**
**A^42^**	Goa, 2008, Sangath (Indigenous NGO)	Yuva Mitr (“friend of youth” in the Konkani language) was a pilot project to assess the acceptability, feasibility, and potential effectiveness of a multicomponent, population-based intervention in improving a range of priority health outcomes for youth aged 16–24 years in urban and rural communities in Goa. Quasi-experimental: pre- and post-test design comparing intervention and control groups.	**Inputs** *Intervention*: Multi-component informational package, along with health information materials. *Target population*: Young people aged 16 to 24 in both rural and urban areas. *Human resources*: One psychologist, two social workers, teachers and three peer educators. **Process** The intervention package was delivered by peer educators and teachers, both of which were trained and equipped with health information materials collated from existing resources. This intervention package consisted of delivering information on emotional health, self-harm, substance use, reproductive and sexual health, violence, and help seeking for health problems. Peer leaders were recruited and trained to provide information to other youth in their communities. Teachers in educational institutions were trained on effective teaching methods; strategies to improve teacher–student relationships; detection and management of common problems faced by youth in school settings; and counselling skills. In the urban community of Margao, four wards were randomly selected as the intervention community, and the remaining six were the comparison community. In the rural community, the largest village, Barcem, was randomly chosen as the intervention community and the remaining three were the comparison community. The three peer educators delivered the intervention with support from the Community Advisory Board (CAB), which is comprised of key people such as village council leaders in the rural community, and with support from trained teachers in the urban community. The duration of the intervention was 12 months. Effectiveness was assessed through a survey at baseline and a follow-up survey 18 months later. Outcomes were measured using a structured interview questionnaire with all eligible youth.	**Outputs** *Coverage*: Peer educators reached 767 youth in the rural community out of 2,232 eligible youth; the coverage in the urban community was not specified in the report. Quality assessment: The quality of intervention delivery was monitored through on-site supervision and weekly review meetings. No results of quality assessment were reported. **Outcomes** *Rural community* Perpetration of physical violence (OR=0.29, 95% CI: 0.15–0.57), menstrual problems (OR=0.39, 95% CI: 0.26–0.60) decreased significantly in the intervention group (p<0.01). Help seeking for reproductive and sexual health (RSH) complaints by women (OR=2.09, 95% CI: 1.07–4.06) increased significantly in the intervention group (p<0.01). Knowledge and attitudes about RSH (OR=1.55, 95% CI: 1.06–2.28) increased significantly in the intervention group (p<0.01). *Urban Community* Experience of sexual abuse (OR=0.19, 95%CI: 0.09–0.41, p <0.001) and perpetration of physician violence (OR=0.59, 95%CI: 0.40–0.87, p 0.01) decreased significantly in the intervention group. Complaints of vaginal symptoms (OR=0.49, 95% CI: 0.26–0.93, p 0.03) and complaints of penile discharge (OR=0.36, 95% CI: 0.24–0.55, p <0.001) decreased significantly in the intervention group. Knowledge and attitudes about RSH (OR=1.46, 95% CI: 1.09–1.97) increased significantly in the intervention group (p 0.01).	Use of self-reported data Probability of observer bias, as researchers were not blind to the allocation status of the community Lack of power and adjustment for clustering may have led to exaggerated results
**B^43^**	Chandigarh, 2005, Postgraduate Institute of Medical Education and Research (PGIMER) (Academic Institution)	To develop a reproductive health education package for adolescent girls of Chandigarh, and to evaluate its effectiveness in improving their knowledge and perceptions about reproductive health when delivered through different health educational strategies like peer education and conventional education. Quasi-experimental: pre- and post-test design comparing intervention and control groups.	**Inputs** *Intervention*: Reproductive health package for adolescent girls delivered through peer education or educational sessions in schools. *Target population*: Adolescent girls aged 15 to 19 years old in classes X, XI, XII in three randomly selected Government Girls Senior Secondary Schools of Chandigarh. *Human resources*: Public health nurse and peer educators. **Process** The contents of the reproductive health education package consisted of anatomy and physiology; puberty and menstruation; conception and contraception; nutrition; immunizations; legal provisions relating to child marriage and pregnancy termination; and sexually transmitted diseases including HIV. The package was designed in consultation with parents, teachers and adolescents; a guidebook was provided to educators and self-reading materials were provided to the target groups. The delivery of the materials was done by 20 trained, supported peer educators and one public health nurse in two government schools. The peer educators communicated messages to their peers, whilst the nurse held 15 educational sessions over a period of nine months with three groups of 30 - 35 students. A third school acted as the control group. The duration between the baseline and the follow-up survey was 11 months.	**Outputs** *Coverage*: Educational sessions reached 95 (out of 100) adolescents vs peer educator approach reached 84 (out of 100) adolescents. *Quality assessment:* No results of quality assessment were reported. **Outcomes** Reproductive health knowledge scores improved significantly after intervention through educational sessions (27.28) and peer education (20.77) in comparison to the control groups (3.64). Knowledge about pubertal changes significantly increased in both the educational session and peer education groups (p<0.05) as compared to the control group. Knowledge about maternal and child health and family planning significantly increased in in both groups after the sessions (p<0.05) as compared to the control group. Knowledge about reproductive tract infections, sexually transmitted infections and HIV increased in both educational sessions and peer education group after the sessions (p<0.05) as compared to the control group.	Results are not generalizable to all adolescent groups e.g. adolescent boys, adolescents living in rural areas and non-school going.
**C^44^**	Uttar Pradesh, 2002, CARE India (India chapter of international NGO)	Foster the development of alternative socialization processes for adolescent girls that enhance the development of positive sexual and reproductive health behaviours. Integrate vocational counselling, training, and follow-up support for adolescent girls coupled with encouragement of savings formation into CARE’s Action for Slum Dwellers’ Reproductive Health project in Allahabad. Increase participation by adolescent girls in other reproductive health-related activities of the Action for Slum Dwellers’ Reproductive Health, Allahabad (ASRHA) Project (e.g., sexual health, hygiene, and nutrition). 4. Foster community acceptance of physical mobility by adolescent girls, strengthen and enlarge positive peer-to-peer support networks, and develop new mentor relationships between younger and older women. Quasi-experimental: pre- and post-test design comparing intervention and control group.	**Inputs** *Intervention*: Multi-component package offering livelihoods, savings formation, reproductive health and communication skills. *Target population*: Adolescent girls between the ages of 14 and 19 years residing in urban slums of Allahabad. *Human resources*: Study team, consultants, AGGs (adolescent girl guides/peer educators) and AGG assistants . **Process** The reproductive health training sessions were conducted using a set of five specially designed flipbooks in Hindi that related to the experiences of a 12-year-old girl. The flipbooks covered the following topics: physiological and behavioural changes during puberty; menstruation; pregnancy and birth; age at marriage and birth spacing; and family planning, including husbands’ roles. The AGGs were responsible for forming groups of peers from their communities and for conducting reproductive health sessions, which were held at their own residences. Each flipbook was completed in 1-2 hours, depending on the girls’ participation. In addition to reproductive health information, AGGs provided counselling regarding vocational training and savings formation. The study team was present at the meetings to help the AGGs educate the girls. In addition, eight assistant peer educators were trained to support the AGGs during the intensive group work. The effectiveness of the intervention was assessed with a follow-up survey conducted 8 months after the baseline survey.	**Outputs** *Coverage*: Information on coverage was not mentioned. *Quality assessment:* No results of quality assessment were reported. **Outcomes** Increase in adolescent girls’ knowledge of reproductive health between baseline and follow-up surveys, including: being able to correctly name one or more contraceptive methods (89% to 97%), being able to name an STI (67% to 94%), being able to correctly answer a question about duration of pregnancy (76% to 100%), and knowing sexual contact was required to become pregnant (44% to 98%).	High lost to follow-up rate; 62 cases matched between baseline and midline
**D^45^**	Uttar Pradesh (UP) and Delhi National Capital Region (NCR), 2015, The YP Foundation (Indigenous NGO)	Scale 1760 young people’s knowledge of and access to Sexual and Reproductive Health and Services and Life Skills by implementing programs on the same through a peer-to-peer approach at community levels with both in and out-of-school young people. Strengthen the demand for Sexual and Reproductive Health Services at village, block and district levels, using existing service delivery platforms through community sensitization for adolescent and youth friendly preventive, promotive and curative services. Advocate for implementation of peer education both in school and out of school at the state level in Uttar Pradesh and advance National Level dialogue and policy advocacy with key civil society actors, UN agencies and government ministries. Descriptive.	**Inputs** *Intervention*: Comprehensive sexuality education (CSE) and community outreaches. *Target population*: Young people across two states in 14 villages across Jhansi and Lucknow districts in UP and 12 low-income sites in Delhi NCR. *Human resources*: Trained peer educator and YP staff members. **Process** The CSE curriculum covered a range of topics including gender and sex; anatomy; puberty, menstruation; masturbation; relationships, negotiation and consent; violence; sexuality; conception, contraception and abortion; and sexually transmitted infections, including HIV. Its development built on existing best practices for those aged 10 to 14 and 15 to 18. The sessions were designed to increase participants’ knowledge, to encourage critical thinking about gender, to affirm body-positive attitudes and to learn how to exercise their rights. The curriculum was 25 hours in total and was imparted by peer educators during 2-hour long, weekly workshops to young people. A total of 400 workshops were conducted by peer educators both in-school and out-of-school settings in partnership with 22 civil society organisation (CSOs). In addition to CSE, peer educators and partnering CSOs planned their strategies for outreach to marginalized communities to create demand. In Jhansi, the intervention included puppet shows, street theater and a district-level dialogue and rally. In Lucknow, street theater, district-level dialogue with stakeholders and 1-to-1 meetings with service providers were utilized. In Delhi NCR, the intervention included role-plays, poster making, film screenings, engagement of participants with peers on specific issues such as HIV/AIDS, healthy relationships and domestic violence.	**Outputs** *Coverage*: CSE workshops were conducted with 914 participants in Delhi NCR and 1,154 participants across Jhansi and Lucknow. No information on denominators was mentioned. *Quality assessment:* No results of quality assessment were reported. **Outcomes** Increased knowledge on sexual reproductive health information and services amongst 1750 young people in the NCR, Lucknow and Jhansi – no metrics were provided in the report. Increase in community sensitization for adolescent and youth friendly preventive, promotive and curative services in Delhi NCR, Lucknow and Jhansi. Increase in advocacy for implementation of peer education both in school and out of school.	Accurate change in participant knowledge difficult to capture due to young participants’ unfamiliarity with the issues in survey questions
**E^46^**	West Bengal, 2015, CINI (Indigenous NGO)	*Out of School: Link Worker Scheme* Increase knowledge and skills on SRH including gender, HIV and AIDS, RTI/STI, correct contraceptive usage and safe abortion services among young people. Improve knowledge on Pre-Conception and Pre-Natal Diagnostic Techniques Act (PCPNDT Act, 1994). Increase in consistent and correct condom use among young people to reduce STI and HIV transmission. Contribute to reduced unplanned pregnancies and unsafe abortions. *In School: Health Program* Build capacity of government school teachers on Life Skill Education (LSE) including SRH and nutrition. Increase knowledge, life skills and awareness on SRH and nutrition rights and services (including related to HIV and AIDS and gender) for adolescent girls. Provide technical support to Government of West Bengal for integrating LSE in school-based curriculum linked to right to education, leading to sustainable and scalable framework. Quasi-experimental: pre- and post-test design.	**Inputs** *Intervention*: Multicomponent intervention to advance young people’s sexual and reproductive health and rights through government - civil society partnership. *Target population*: Adolescents in school and out of school in 4 districts of West Bengal. *Human resources*: Link workers, CINI team members, block and district level health functionaries including Anwesha counsellors (lady health counselors at adolescent health clinics), school teachers and peer educators. **Process** The topics covered in both out of school and in school interventions included: adolescence; puberty; nutrition; and key SRH issues such as reproduction, contraception, safe abortion, reproductive tract and sexually transmitted infections and HIV. Trained peer educators were involved in disseminating this information through both the in-school and out-of-school components of the programme and making linkages with services for young people. The out-of-school link workers scheme had a focus on reaching out to vulnerable adolescents in rural areas through peer educators, whilst the School Health Programme focused on building the capacities of school teachers to delivery Life Skills Education (LSE) in addition to peer education. CINI’s interventions also included project sensitization meetings with key stakeholders such as functionaries of the Ministry of Health and Family Welfare (MoHFW), Integrated Child Development Services (ICDS) and the Education Department. Health system functionaries such as counselors, block-level medical officers, auxiliary nurse midwives and accredited social health activists (ASHAs) were also included in such meetings to in order to enhance linkages with services. Additionally, the project capitalized on events (e.g. World AIDS Day), health fairs and Village Health and Nutrition Days to galvanize local communities. The effectiveness of CINI’s intervention was assessed through an endline survey conducted 3 years after the baseline survey.	**Outputs** *Coverage:* Out of school: Peer educators reached out to 10,000 adolescents and young people across 200 villages in 2 districts; no information on the denominator was provided In school: Peer educators reached out to 3,824 students directly who, in turn, reached out to another 123,407 indirectly across the 2 districts; no information on the denominator was provided. *Quality assessment*: During the implementation CINI team members conducted process documentation, but no results were included in the report. No results of quality assessment were reported. **Outcomes** *Out of School:* Increases in knowledge were reported for – Contraceptives as protection against pregnancy (Burdwan district: 34% to 87%; North Dinajpur: 22% to 76%) Prevention of HIV & AIDS (Burdwan district: 73% to 78%; North Dinajpur: 49% to 87%) HIV testing (Burdwan district: 29% to 83%; North Dinajpur: 20% to 91%) Legal age at marriage for girls (Burdwan district: 29% to 92.5%; North Dinajpur: 23% to 94.5%) Legal age at marriage for boys (Burdwan district: 26% to 77%; North Dinajpur: 52% to 86%) *In School:* Increase in knowledge was reported for – Contraceptives as protection against pregnancy (South 24 Paraganas: 32% to 71.5%; Murshidabad: 22% to 72.5%) Prevention of HIV & AIDS (South 24 Paraganas: 55% to 73%; Murshidabad: 80% to 82%) HIV testing (South 24 Paraganas: 50% to 78.5%; Murshidabad: 75% to 87%) Legal age at marriage for girls (South 24 Paraganas: 29% to 95%; Murshidabad: 30% to 96.5%) Legal age at marriage for boys (South 24 Paraganas: 60% to 94.5%; Murshidabad: 71% to 94.5%)	*Out of School* Deep rooted social norms and taboos related to discussion on SRH issues and participation of girls in activities took time to transcend. Link workers working at fixed remuneration for many years, affecting motivation . *In School* Hesitation among school authorities and teachers in dealing with SRH content particularly in co-ed schools. Lack of sufficient teachers in schools impacting workload of Master trainers; also their varying backgrounds and interest levels. High dependence on CINI to implement the peer education.
**F^47^**	West Bengal, 2016, CINI, Indigenous NGO)	To strengthen the implementation of the Adolescent Health Strategy [RKSK] of Government of India to contribute to the overall development of the adolescents in the state of West Bengal. To build capacity of adolescent girls and boys on reproductive sexual health and rights, hygiene, nutrition, non-communicable diseases, substance misuse, psychosocial health, gender-based violence free lifestyle through strengthening convergence between different Government adolescent development programs of different Departments like Health and Family Welfare, Women and Child Development and Education. To empower adolescent girls and boys on technical aspects like identifying vulnerability issues, planning, and leadership so that they can participate in local convergence platforms. To enhance technical capacity of service providers and local self-government to lead a sustainable process of adolescent development in their area through convergence between different programs for adolescents. Evidence-based documentation of the holistic, scalable and sustainable model of a common adolescent development program to be replicated at state and national levels through Government-Civil Society partnership. Descriptive.	**Inputs** *Intervention*: Sensitization on the six RKSK components . *Target population*: Adolescent girls and boys aged 10 to 19 years living in four community development blocks of two resource poor districts in West Bengal: South Twenty-four Parganas and Murshidabad in the State of West Bengal . *Human resources*: CINI staff members, peer educators, peer educator master trainers from local NGOS. **Process** The six components of RKSK are: nutrition, sexual and reproductive health (SRH), non-communicable diseases (NCDs), substance misuse, injuries and violence (including gender-based violence) and mental health. Over the period of a year, peer leaders conducted interactive sessions, trainings and role plays as well as shared information, education and communication materials to impart knowledge about SRH adolescents in their respective localities. Each peer leader was assigned 10 adolescents and were given tracking sheets to monitor ‘risky’ behaviour, health practices and problems. A mix of qualitative (focus group discussions, in-depth interviews) and quantitative research (close-ended questionnaire) was used for impact assessment. The impact assessment was conducted 10 months after the implementation of the intervention.	**Outputs** *Coverage*: 136,153 (male = 61,507; female= 74,646) adolescents were sensitized about the RKSK components, from a desired aim of reaching 180,000 adolescents. *Quality assessment*: The sustainability of the project was ensured by monitoring the activities of peer leaders regularly. A community monitoring mechanism was also developed that involved administrative officials, health officials, parents, teachers and adolescents and together they keep a vigil on adolescents in the locality. No results of quality assessment were reported. **Outcomes** More than 5,585 cases referred to the Adolescent Friendly Health Clinics by peer leaders and master trainers to access counselling and condoms Improved SRH awareness and practices among adolescents Improvement in SRH awareness and practices, namely related to onset of menstruation; menstrual hygiene; prevention SRH related ailments; maintaining personal hygiene and eating nutritious food; and significant increase in in use of sanitary napkins Increased reporting of SRH-related issues by adolescents, including higher reporting of menstrual problems from adolescent girls	Need for support in terms of refresher trainings on adolescent topics as there was a general feeling of “not knowing enough”. Need for aids to conduct sessions – video, IEC and other interactive materials so as to make the knowledge transfer better and uniform. Insufficient training for peer educators. Lack of resources for standardized high-quality knowledge transfer.
**G^48^**	Uttar Pradesh (UP), 2003, Population Council (International NGO) and CARE India (India chapter of international NGO)	To examine whether an experimental intervention for girls aged 14–19 that provided vocational counselling and training and assistance with opening savings accounts in slum areas of Allahabad. Increased physical mobility and contact with individuals outside the family as well as awareness of safe places for girls to congregate. Increased self-efficacy. Increased reproductive health knowledge. Altered work aspiration and encouraged more progressive gender role norms. Reduced time spent on domestic tasks and increased time spent on productive tasks. Quasi-experimental: pre- and post-test design comparing intervention and control groups.	**Inputs** *Intervention*: Multicomponent intervention including provision of reproductive health information, vocational counselling and training, and assistance with opening savings accounts. *Target population*: Adolescent girls 11 to 18 years of age residing in the urban slums of Allahabad. *Human resource*s: Staff members of CARE, Population Council, and the Centre for Operations Research and Training (CORT), as well as peer educators. **Process** The intervention, which began in 2001, integrated livelihoods activities for adolescent girls aged 14 to 19 years into CARE’s reproductive health program for slum dwellers known as ‘Action for Slum Dwellers’ Reproductive Health, Allahabad’ (ASRHA). The reproductive health curriculum used included the following topics: puberty, menstruation, reproductive biology, pregnancy, contraception, sexually transmitted infections and age at marriage. Each trained peer educator was expected to visit every household in her locality and invite all eligible girls (11 - 18 years old) to participate in the project. When approximately 20 girls were ready to participate, a group was formed and met at the home of a peer educator. Participants residing in the experimental slums received the reproductive health training sessions and follow-up support from a peer educator. The vocational counselling and savings components were provided by project staff after the completion of the reproductive health curriculum and were open only to those participants who had maintained good attendance and were in the 14 to 19 years age bracket. CORT conducted the census of the selected slums and data collection for baseline and endline surveys. The duration between the baseline and endline survey was 3 years.	**Outputs** *Coverage*: Peer educators reached 525 adolescent girls out of 1,683 eligible adolescent girls interviewed at baseline. *Quality assessment*: No results of quality assessment were reported. **Outcomes** There were significant improvements in the following: Increase in knowledge of safe spaces for girls: intervention 83.2% vs. control matched 33.7% (p ≤0.001) Increase in reproductive health knowledge (mean score): intervention 6.7 vs. control matched 5.7 (p ≤0.001) Increased social skills (mean) and group membership: intervention 12.0 vs control matched 11.0 (p ≤0.05) and intervention 15.6% vs. control matched 5.1% (p ≤0.01) respectively Increased time spent on leisure activities (mean): intervention 4.4 vs control matched 3.7 (p ≤0.05) There were no significant effects found on gender-role attitudes, mobility, self-esteem, work expectations, or on number of hours visiting friends, performing domestic chores, or engaging in labor-market work.	Difficult to conduct a longitudinal survey in an urban slum area – unable to match participants from baseline to endline. Intervention duration and intensity insufficient to produce sizeable effect. Outcome variables used to evaluate the impact of the intervention were not appropriate.
**H^49^**	Two districts of Jharkhand – Gumla and Ranchi, 2013, The Centre for Development and Population Activities (CEDPA) (Indigenous NGO)	To examine CEDPA India’s role in the provision of technical assistance at the state level and experiences in implementation of the SABLA scheme in Gumla as a case study and recommend a framework for scaling up of the scheme at the national level. To assess change among select adolescent girls at the personal level. To assess innovations brought in by CEDPA India for effective implementation of the scheme and adequacy, efficiency and effectiveness of the innovations. To assess gaps and challenges in effective implementation of SABLA. To recommend framework for scale up. Quasi-experimental: pre- and post-test design comparing intervention and control group.	**Inputs** *Intervention*: Implementation of SABLA scheme with a focus on nutrition and sexual and reproductive health (SRH). *Target population*: Adolescent girls aged 11 to 18 years. *Human resources*: Staff from CEDPA India, child development project officers at the district level, lady supervisors, local NGOs, and peer educators (sakhis [peer monitor]/sahelis [peer leaders]). **Process** A series of activities were undertaken, including a baseline survey, to assess the current context of adolescent girls; the training and capacity building of Anganwadi Workers (AWWs), Sakhi and Sahelis; and monitoring of vocational training for the adolescent girls participating in the Scheme. SABLA helped implement the scheme in two districts of Ranchi and Gumla, with a control group in Bokaro. CEDPA undertook a training at the district level of child development project officers, lady supervisors, and NGOs from both districts. These workers then trained adolescent girls who implemented the scheme at the village level. CEDPA facilitated another training of Sakhis/Sahelis to inform them about SABLA Scheme and train them to conduct sessions in their respective groups (Samuhs). CEDPA developed a ready reckoner for Sakhis/Sahelis, which they and anganwadi workers used regularly to update their knowledge before a Kishori Samuh meeting. The duration between the baseline and follow-up evaluation was approximately 12 months.	**Outputs** *Coverage*: Not mentioned. *Quality assessment:* CEDPA regularly monitored and provided technical assistance to Sakhi, Sahelis, and Anganwadi workers. They also liaised with the child development project officers and the supervisors on a regular basis*.* No results of quality assessment were reported. [All results are not available as this was a midline report, with only qualitative findings emerging out of it. There was no mention of the impact of the SABLA scheme in the control group]. **Outcomes** Both AWWs and peer educators appreciated the way the training was facilitated and they enjoyed the activities, role plays and games played during the training; they found the program useful and easy to understand Sakhis and Sahelis who underwent the three days’ training reported that it was informative and useful The facilitators and the girls felt that even though the programme is new, participating in SABLA Scheme was a positive experience There was an increase in knowledge and confidence levels amongst the girls, who could negotiate and solve problems and communicate more effectively with their parents Adolescent girls became more aware about various issues like nutrition, menstrual hygiene and reproductive health	No clear-cut guidelines or uniformity in the organisation of Kishori Samuh meetings. AWWs find it difficult to dedicate time to Kishori Samuhs due to the multitude of responsibilities entrusted to them under the ICDS programme. Permissions needed for implementation from relevant government officials on an ongoing basis; the process was time consuming.
**I^50^**	Bihar, 2015, Centre for Catalyzing Change (indigenous NGO), Population Council (International NGO), and London School of Hygiene & Tropical Medicine (Academic institution)	1) To measure the impact of the Do Kadam Barabari Ki Ore (Two Steps Towards Equality) project among boys in ages 13–21 years, who were members of youth clubs supported by the NYKS program of the Ministry of Youth Affairs and Sports, in: Changing gender role attitudes, attitudes toward violence against women and girls, and controlling behaviours over sisters, girlfriends, and wives. Reducing the perpetration of various forms of violence against women and girls. 2) To assess the feasibility and acceptability of the intervention Randomized control trial with in-depth interviews and panel surveys.	**Inputs** *Intervention*: Multi-component intervention comprised of gender transformative life skills education combined with cricket-coaching. *Target population*: Adolescent boys aged 13 to 21 years. *Human resources*: Centre for Catalyzing Change (C3) staff members and peer mentors. **Process** Drawing on previous, successful programmes for boys, the Do Kadam intervention focused on promoting egalitarian gender attitudes and abhorrence of violence against women and girls through gender transformative life skills education combined with cricket-coaching. The intervention was delivered over a period of 18 months through 42 weekly sessions, discounting festivals and holidays. One hour each week was devoted to gender transformative life skills education, which was delivered by peer mentors, and one hour to cricket-coaching delivered by C3 staff members. Peer mentors used the training module to deliver each session, using the guidelines and participatory methodologies recommended for each session. C3 core trainers supported peer mentors to ensure that sessions were conducted as per the guidelines, that questions raised by participants were answered adequately, and that peer mentors were able to maintain discipline during the sessions. Participants were surveyed both at baseline and at endline. The duration between the two surveys was 2 years.	**Outputs** Coverage: Peer mentors reached out to 516 boys in 15 youth clubs in the intervention group out of 583 eligible unmarried boys interviewed at baseline. *Quality assessment*: No results of quality assessment were reported. **Outcomes** Boys in the intervention arm expressed egalitarian gender role attitudes and notions of masculinity in 6.6 of 9 attitudes probed, compared to 6.2 reported by those in the control arm (effect estimate 0.40, p ≤0.05) Boys in the intervention arm rejected men’s and boys’ right to exercise control over women in 4.9 situations, compared to 4.2 situations reported by those in the control arm (effect estimate 0.7, p ≤0.01) Of 17 situations probed, boys from the intervention arm rejected the right of men and boys to exercise violence against women and girls in 11.7, compared to 10.3 among those in the control arm (effect estimate 1.3, p ≤0.001) In terms of perpetration of various forms of gender-based violence, there was weak evidence that non-contact forms of violence, such as stalking a girl, had declined because of the intervention. However, in in-depth interviews, several boys’ narratives suggested at the endline interview that they no longer participated in teasing girls and some of them specifically attributed the change they had experienced directly to what was conveyed in the Do Kadam programme.	Number of sessions too few to identify change Peer mentors lacked confidence and communication skills, and were uncomfortable conveying sensitive messages such as those related to sexual violence
**J^51^**	Maharashtra, 2008, Albert Einstein College of Medicine, Yeshiva University, New York and Drug Abuse Information, Rehabilitation and Research Center, Mumbai (Academic Institution and Indigenous NGO)	To test whether an educational program built on specific cultural, linguistic, and community-specific characteristics was effective in improving knowledge, beliefs, and attitudes about HIV infection in general and increased confidence in youth (8 grade students, 13-21 year old) in dealing with high-risk situations. Quasi-experimental: pre- and post-test design comparing intervention and control group	**Inputs** *Intervention:* School-based Teenage Education Program (STEP) focusing on HIV Prevention. *Target population:* Eighth grade students (13-15 years old) in 25 schools randomly selected in Mumbai. *Human resources*: Peer educators, staff members from implementing organisation (DAIRRC). **Process** The educational programme was developed by combining curriculum from successful HIV prevention programs in the United States with existing curriculum-based drug education efforts modeled on the social learning theory of Drug Abuse Information, Rehabilitation and Research Center (DAIRRC). The programme was developed to improve knowledge, beliefs and attitudes about HIV infection and increase young people’s confidence in dealing with high-risk situations. Trained peer educators conducted the STEP program over 6 weeks (single one-hour session per week for six consecutive weeks) in participating schools. Two classes in each school participated, and the school administrator randomly assigned the classes to intervention and control arms. Students in the intervention group were exposed to the STEP curriculum, while those in the control arm received no curriculum. The baseline survey was completed by 1,846 students (946 intervention and 900 control), and the endline survey was completed by 1,733 (882 intervention and 859 control). The duration between the baseline survey and endline survey was 6 weeks.	**Outputs** *Coverage*: 1800 students in 25 Mumbai schools. *Quality assessment*: No results of quality assessment were reported. **Outcomes** Knowledge of HIV/AIDS: Students who received STEP curriculum evidenced greater knowledge after 6 sessions (p≤.001); girls had lower knowledge at baseline but significantly higher compared to boys at the end of intervention (p<.05). Beliefs: change in belief towards more tolerance, more pronounced in the intervention group (p≤.001; both genders reported improved positive beliefs towards people living with HIV/AIDS (p<.001). Attitudes: difference in attitudes significant in the intervention group for four attitudes - abstinence (p<.01), using condom consistently (p<.001), understanding of precautions (p<.05), less likely to be influenced by peers (p<.001). Confidence: assessed refusal skills in dealing with peer and social pressure. Differences more significant and pronounced for intervention group (p≤.001).	Most trainers in the program were females. Intervention and control groups were in the same school. Findings are not generalizable to rural area, as intervention took place in metropolitan area.
**K^52^**	Maharashtra and Uttar Pradesh, 2008, Committee of Resource Organizations (CORO) for Literacy, MAMTA and DAUD (International and Indigenous NGOs)	To reduce HIV vulnerability among men and women and to reduce young men’s use of violence against women and girls through the promotion of gender equitable attitudes and behaviours. To adapt the Gender Equitable Men (GEM) Scale, initially tested and developed in Brazil, to the Indian context for use as an evaluation tool. To test the impact of peer-led group educational activities and community-based social marketing campaigns in promoting gender equitable attitudes and behaviours and safe sexual practices among young men from low-income communities in Mumbai. Adapt and test the effectiveness of peer-based group educational activities among young men in rural settings in Gorakhpur, Uttar Pradesh. Quasi-experimental: pre- and post-test design comparing intervention and control group.	**Inputs** *Intervention*: Group education sessions (GES) and lifestyle social marketing campaign (LSSM) delivered through peer education, collectively referred to as ‘Yaari-Dosti.’ *Target population*: married and unmarried men age 15-29 in urban and rural settings from Mumbai, Maharashtra and Gorakhpur, Uttar Pradesh respectively. *(Note: There is no information on the proportion of 24-29 year olds. We included the study as it was a well written report showcasing knowledge, attitudinal and behavioural outcomes.)* *Human resources*: CORO for Literacy and MAMTA staff members in Mumbai and Gorakhpur and peer educators. **Process** The themes covered through the GES were: gender and sexuality: STIs and HIV risk and prevention; violence; reproduction; alcohol and risk; and HIV-related stigma and discrimination. The topics were covered using participatory learning methods, including games and role-playing, that engaged the participants in discussion, debate and critical thinking. The GES were implemented over 6 months (one session per week) by peer educators, each session lasting for over one hour. Facilitators and field supervisors, together with gender experts, met once every month to discuss and share experiences about implementation. The LSSM component reinforced messages from the GES and focused on gender-equitable lifestyles and versions of manhood through community-based activities conducted in spaces where young men congregate. This was delivered through street plays; posters; and distribution of pamphlets, comic strips, community-based discussions, t-shirts and condoms at a ‘mobile booth.’ The LSSM campaign was developed and led by peer educators using information gathered during the formative research phase and subsequent testing of messages in the community. The campaign developed materials based on young men from these communities that projected an alternative form of masculinity. The effectiveness of the intervention was assessed through a survey at baseline and follow-up survey approximately 6 months later.	**Outputs** *Coverage*: Peer-educators reached out to 1,178 peers out of 1,195 young men in the chosen communities. *Quality assessment:* No results of quality assessment were reported. **Outcomes** A total of 1,195 (875 Mumbai + 1040 Gorakhpur) completed the baseline survey and 1,138 (537 Mumbai + 601 Gorakhpur) completed the follow-up survey. Some of the notable outcomes were: Communication with partners on condoms, sex, STIs, and/or HIV significantly improved in the intervention sites. Improvements among the intervention participants in discussing key reproductive and sexual health issues (condom use, sexual relationships, STIs, and HIV/AIDS) with a female partner in the last three months (p <0.05). There was a significant increase in condom use at last sex with all partner types in the intervention areas (p <0.05). Men in the intervention arms were 1.9 times more likely to have used condoms at last sex in Mumbai (p <0.001) and 2.8 times more likely to have used them in Gorakhpur (p <0.001) than those in the comparison arms in each setting. Report of sexual health problems during the previous three months decreased significantly in the intervention sites from baseline to follow-up (p <0.05). There was a positive trend toward improvements in GEM Scale scores being associated with decreases in HIV/STI risk behaviours (not significant). Young men in the intervention sites reported more positive attitudes toward person living with HIV (p <0.05).	Self-selection of the study participants. Outcome measures relied on self-reports of participants.
**L^53^**	12 states: Uttar Pradesh, Haryana, Punjab, Bihar, Odisha, Maharashtra, Rajasthan, Karnataka, Andhra Pradesh, Tamil Nadu, Assam, Tripura, 2013, Administrative Staff College of India (Academic institute)	To evaluate the Rajiv Gandhi Scheme Empowerment Adolescent Girls – Sabla. To assess the scheme from the perspective of functioning of the scheme and all its components including nutrition component; non-nutrition component; administrative component; flow of funds; training component; publicity component; adolescent girls being serviced through the scheme; role of government officials (State level, district level, block level and village-level) their response towards the scheme; non-beneficiaries; and other stakeholders including families of the AGs and PRI members, Community leaders of the village. To assess the strengths of the scheme and barriers against the scheme. To provide a ‘way forward’ for the scheme. Descriptive.	**Inputs** *Intervention*: Multi-component scheme comprised of nutrition and non-nutrition educational sessions, life-skills sessions and vocational training. *Target population*: Adolescent girls 11 to 18 years of age. *Human resources*: Anganwadi workers (AWW), AWHs, department of health officials, NGOs, self-help groups, Child Development Project Officer, adolescent girl peer monitors and educators. **Process** This multi-component intervention focused on improving the health and nutrition status of adolescent girls by raising their awareness about health, hygiene, nutrition, adolescent reproductive and sexual health (ARSH), family and childcare. Adolescent girls also received life skills education and vocational training. The nutrition component of the scheme was delivered by AWWs, who also supported the peer monitors (sakhis) and peer leaders (sahelis) to deliver the life skills and vocational trainings in places where adolescent girls receive information. Adolescent girls also made social connections with their peers; built confidence and morale; and received support for envisioning their futures through the scheme. The evaluation relied on a mix of quantitative and qualitative methods. It was designed to be conducted in 12 states distributed across the five regions (at least 2 from each) of the country over a two-year period. The selection of states was based on the pilot districts and states where the scheme had been implemented. The fieldwork phase included field survey with beneficiaries conducted largely at anganwadis or in a school hall; focus group discussions with beneficiaries and with community members; and key informant interviews with officials, staff and others.	**Outputs** *Coverage*: coverage of nutrition component was 1.13 crores beneficiaries out of a target of 10,170,443; coverage of family welfare, SRH and childcare practices increased from 3,592,506 adolescent girls in 2011-12 to 4,811,264 adolescent girls in 2012-13. *Quality assessment:* In order to ensure effective implementation and monitoring of the Sabla throughout the country, a National Monitoring and Supervision Committee has been set up under the chairpersonship of the Secretary, Ministry of Women & Child Development. This Committee meets quarterly or as and when required at the notice of the Chairperson. No results of quality assessment were reported. **Outcomes** 71.6% of the total respondents had ever attended ARSH counselling out of 3,358 out of school girls surveyed, Out of out of 2,322 adolescent girls surveyed: 96.4% AGs reported that the counselling sessions were useful or very useful 70.9% of the AGs reported having some knowledge about HIV/AIDS 87% claimed that they would visit a doctor when pregnant. 75.2% of respondents were aware of Kishori Samooh Around 85.2% sakhis and sahelis (peer mentors) reported a sense of ownership of the delivery of activities under the Sabla scheme out of 424 and 780 sakhis and sahelis surveyed.	In the absence of baseline, and given the short span since program implementation, and the short study timeline, the impact questions from the beneficiary perspective could not be fully investigated such as nutritional impacts or behavioural and change in practices. Ineffectiveness of the anganwadi workers in motivating the adolescent girls to attend the anganwadi regularly. Limited infrastructure in AWWs to organise Kishori meetings. Improper reporting of scheme components and difficulty in filling forms and consolidating data was observed. Low involvement of sakhis and sahelis in the scheme.
**M^54^**	Jharkhand, 2015, Centre for Catalyzing Change (C3) (Indigenous NGO)	To increase the acceptability of and access to family planning methods by married adolescent couples by providing knowledge and information on modern contraceptive methods to enable them to plan and limit their family size, in Ramgarh district of Jharkhand, India. To ensure effective linkages between eligible couples and service providers for accessing Reproductive Health Services including Family planning services. Quasi-experimental: pre- and post-test design.	**Inputs** *Intervention*: Information on reproductive health practices such as family planning and accessing reproductive health services. *Target population*: Married adolescent couples – girls (10-19 years) and their husbands (14-25 years) residing in Ramgarh district. *Human resources*: Project personnel from C3 team and Nav Bharat Jagrity Kendra, 50 trained peer educators. **Process** The intervention was designed to increase knowledge and awareness of age of marriage, reproductive health practices, delaying first pregnancy, use of family planning methods, linkages with service providers, and access and availability of reproductive health services in the health facilities. A total of 3,038 meetings (2 meetings/fortnight/village/month) were conducted during the period with married adolescent couples in 50 target villages. The peer educators gave the scheduled sessions among the married adolescent couples with support from health facilitators, who are functionaries of the Department of Health and Department of Women and Child Development. The group meetings were organised and held separately for the wives and the husbands due to their availability. The effectiveness of the intervention was assessed through a survey at baseline and an endline survey 12 months later.	**Outputs** *Coverage*: 950 married adolescent couples were identified and reached from approximately 50 villages (25 in each block – Ramgarh Sadar and Patratu); no information on denominators available. *Quality assessment:* No results of quality assessment were reported. **Outcomes** *Knowledge:* Increase in awareness among married adolescent girls (MAGs) e.g. for: contraceptive pills (Baseline: 20%, Endline: 91%), condoms (Baseline: 26%, Endline: 91%), injectable contraceptives (Baseline: 10%, Endline: 36%). Increase in awareness among husbands on married adolescent girls (HMAGs) e.g. for: condoms (Baseline: 39%, Endline: 97%), vasectomy (Baseline: 15%, Endline: 56%), tubectomy (Baseline: 16%, Endline: 59%), contraceptive pills (Baseline: 31%, Endline: 69%), Emergency Contraceptive Pills (Baseline: 8%, Endline: 38%). Increase in awareness of pregnancy detection kit (Baseline: 28%, Endline: 86%) and awareness of its use as well (Baseline: 20%, Endline: 56%). Increase in awareness of complication in pregnancy and childbirth often leading to maternal death as a consequence of early pregnancy: among MAGs (Baseline: 67%, Endline: 87%) and HMAGs (Baseline: 74%, Endline: 96%). Improved knowledge of AFHC clinic both among MAGs and HMAGs. Increase in awareness of benefits provided by Government in case of institutional delivery amongst: MAGs: free transportation to and from the health facility (Baseline: 21%, Endline: 100%), free stay at the health facility (Baseline: 23%, Endline: 100%), free medicines (Baseline: 30%, Endline: 100%), cash benefit (Baseline: 18%, Endline: 100%) HMAGs: free transportation to and from the health facility (Baseline: 36%, Endline: 97%), free stay at the health facility (Baseline: 38%, Endline: 99%), free medicines (Baseline: 47%, Endline: 99%), cash benefit (Baseline: 34%, Endline: 98%) Increase in awareness for ANC, delivery and PNC services for women amongst: MAGs: dietary supplement (Baseline: 66%, Endline: 100%), TT immunization (Baseline: 67%, Endline: 100%), iron folic acid tablets (Baseline: 68%, Endline: 100%), ANC checkup (Baseline: 68%, Endline: 100%), delivery (Baseline: 64%, Endline: 100%), PNC (Baseline: 64%, Endline: 99%) HMAGs: dietary supplement (Baseline: 89%, Endline: 99%), TT immunization (Baseline: 89%, Endline: 99%), iron folic acid tablets (Baseline: 85%, Endline: 98%), ANC checkup (Baseline: 85%, Endline: 99%), delivery (Baseline: 86%, Endline: 99%), PNC (Baseline: 76%, Endline: 96%) *Attitude:* Increase in preference to visit a health facility for symptoms like vaginal discharge, burning micturition, genital itching, genital ulceration and pain in lower abdomen among MAGs (Baseline: 46%, Endline: 93%). Increase in preference to visit a health facility for symptoms like vaginal discharge, burning micturition, genital itching, genital ulceration and pain in lower abdomen among HMAGs (Baseline: 51%, Endline: 92%). *Behaviour:* MAGs reported increase in being advised by ASHA/ANM on delaying first pregnancy (Baseline: 8%, Endline: 55%). HMAGs reported being advised by ASHA/ANM to delay the first pregnancy (Baseline: 2%, Endline: 18%). Increase in using a contraception method (Baseline: 14%, Endline: 59%). Increase of MAGs going to the first point of contact, the local ANM, for confirmation of pregnancy (Baseline 14%, Endline: 75%). Increase in AFHS visits (Baseline: 0% vs Endline: 50%).	Loss to follow-up of MAGs between baseline and endline surveys
