# Supplementary material for: A systematic review of the evidence on peer education programmes for promoting the sexual and reproductive health of young people in India
Source: Sex Reprod Health Matters. 2020 May 6;28(1):1741494. doi: 10.1080/26410397.2020.1741494 (PMC7887991; doi:10.1080/26410397.2020.1741494)
Supplement: Supplementary Box S1 [file ZRHM_A_1741494_SM4283.docx]

**Box S1.** **Types of research study and evaluation designs**

| **Descriptive**: Describes the program characteristics, service utilization, and program processes, outputs, and outcomes without a comparison group.  **Quasi-experimental**: Compares an intervention group to a control group without randomization or compares an intervention group to itself using measurements pre- and post-implementation of program  **Randomized Control Trial**: Compares an intervention and a control group using randomization. |
| --- |

Table S1. Characteristics and main findings of research studies and evaluation reports

| **Location**: This is the state/territory in India in which the study or evaluation was conducted.  **Year**: This is the year that the study or evaluation was completed.  **Organization name and type**: In this column, the name of the implementing and supporting organizations are included alongside a description of the type of organization (e.g. indigenous NGO, international NGO, academic institution).  **Objectives**: The objectives are copied directly from the studies themselves, without making changes to the wording.  **Design**: This column provides information on the type of research study or evaluation used, giving an indication of the authors’ methodology.  **Implementation**: This column provides information on the inputs and process of the study or evaluation. The ‘process’ cell provides a description of the content, delivery and context relevant to the study.  **Results**: This column is divided into outputs (coverage, quality) and outcomes (knowledge, attitudes, practices). Coverage relates to the # of people within the target population reached. Quality relates to assessments done of the peer education (content or delivery). Where available, findings related to all 3 outcomes areas (and any additional ones) are presented with relevant statistics.  **Limitations**: The limitations are taken directly from the studies and are, thus, the limitations and gaps identified by the authors themselves. Limitations relate both to the design of study and the project implementation. | | | | | | | | | | | | | | | | | | |
| --- | --- | --- | --- | --- | --- | --- | --- | --- | --- | --- | --- | --- | --- | --- | --- | --- | --- | --- |
| **ID (ref)** | **Location** | | | **Year** | | | **Organization name and type** | | | **Objectives** | **Design** | | | **Implementation** | | **Results** | | **Limitations** |
| A [42] | Goa | | | 2008 | | | Sangath (Indigenous NGO) | | | Yuva Mitr (“friend of youth” in the Konkani language) was a pilot project to assess the acceptability, feasibility, and potential effectiveness of a multicomponent, population-based intervention in improving a range of priority health outcomes for youth aged 16–24 years in urban and rural communities in Goa. | Quasi-experimental: pre- and post-test design comparing intervention and control groups | | | **Inputs**  *Intervention*: Multi-component informational package, along with health information materials  *Target population*: Young people aged 16 to 24 in both rural and urban areas  *Human resources*: One psychologist, two social workers, teachers and three peer educators  **Process**  The intervention package was delivered by peer educators and teachers, both of which were trained and equipped with health information materials collated from existing resources. This intervention package consisted of delivering information on emotional health, self-harm, substance use, reproductive and sexual health, violence, and help seeking for health problems.  Peer leaders were recruited and trained to provide information to other youth in their communities. Teachers in educational institutions were trained on effective teaching methods; strategies to improve teacher–student relationships; detection and management of common problems faced by youth in school settings; and counselling skills.  In the urban community of Margao, four wards were randomly selected as the intervention community, and the remaining six were the comparison community. In the rural community, the largest village, Barcem, was randomly chosen as the intervention community and the remaining three were the comparison community. The three peer educators delivered the intervention with support from the Community Advisory Board (CAB), which is comprised of key people such as village council leaders in the rural community, and with support from trained teachers in the urban community.  The duration of the intervention was 12 months. Effectiveness was assessed through a survey at baseline and a follow-up survey 18 months later. Outcomes were measured using a structured interview questionnaire with all eligible youth. | | **Outputs**  *Coverage*: Peer educators reached 767 youth in the rural community out of 2,232 eligible youth; the coverage in the urban community was not specified in the report.    Quality assessment: The quality of intervention delivery was monitored through on-site supervision and weekly review meetings. No results of quality assessment were reported.  **Outcomes**  *Rural community*  Perpetration of physical violence (OR=0.29, 95% CI: 0.15–0.57), menstrual problems (OR=0.39, 95% CI: 0.26–0.60) decreased significantly in the intervention group (p<0.01).  Help seeking for reproductive and sexual health (RSH) complaints by women (OR=2.09, 95% CI: 1.07–4.06) increased significantly in the intervention group (p<0.01).  Knowledge and attitudes about RSH (OR=1.55, 95% CI: 1.06–2.28) increased significantly in the intervention group (p<0.01).    *Urban Community*  Experience of sexual abuse (OR=0.19, 95%CI: 0.09–0.41, p <0.001) and perpetration of physician violence (OR=0.59, 95%CI: 0.40–0.87, p 0.01) decreased significantly in the intervention group.  Complaints of vaginal symptoms (OR=0.49, 95% CI: 0.26–0.93, p 0.03) and complaints of penile discharge (OR=0.36, 95% CI: 0.24–0.55, p <0.001) decreased significantly in the intervention group.  Knowledge and attitudes about RSH (OR=1.46, 95% CI: 1.09–1.97) increased significantly in the intervention group (p 0.01). | | Use of self-reported data  Probability of observer bias, as researchers were not blind to the allocation status of the community  Lack of power and adjustment for clustering may have led to exaggerated results |
| B [43] | Chandigarh | | | 2005 | | | Postgraduate Institute of Medical Education and Research (PGIMER) (Academic Institution) | | To develop a reproductive health education package for adolescent girls of Chandigarh, and to evaluate its effectiveness in improving their knowledge and perceptions about reproductive health when delivered through different health educational strategies like peer education and conventional education | | Quasi-experimental: pre- and post-test design comparing intervention and control groups | | | **Inputs**  *Intervention*: Reproductive health package for adolescent girls delivered through peer education or educational sessions in schools  *Target population*: Adolescent girls aged 15 to 19 years old in classes X, XI, XII in three randomly selected Government Girls Senior Secondary Schools of Chandigarh  *Human resources*: Public health nurse and peer educators  **Process**  The contents of the reproductive health education package consisted of anatomy and physiology; puberty and menstruation; conception and contraception; nutrition; immunizations; legal provisions relating to child marriage and pregnancy termination; and sexually transmitted diseases including HIV. The package was designed in consultation with parents, teachers and adolescents; a guidebook was provided to educators and self-reading materials were provided to the target groups.  The delivery of the materials was done by 20 trained, supported peer educators and one public health nurse in two government schools. The peer educators communicated messages to their peers, whilst the nurse held 15 educational sessions over a period of nine months with three groups of 30 - 35 students. A third school acted as the control group. The duration between the baseline and the follow-up survey was 11 months. | | **Outputs**  *Coverage*: Educational sessions reached 95 (out of 100) adolescents vs peer educator approach reached 84 (out of 100) adolescents    *Quality assessment:* No results of quality assessment were reported.  **Outcomes**  Reproductive health knowledge scores improved significantly after intervention through educational sessions (27.28) and peer education (20.77) in comparison to the control groups (3.64)  Knowledge about pubertal changes significantly increased in both the educational session and peer education groups (p<0.05) as compared to the control group  Knowledge about maternal and child health and family planning significantly increased in in both groups after the sessions (p<0.05) as compared to the control group  Knowledge about reproductive tract infections, sexually transmitted infections and HIV increased in both educational sessions and peer education group after the sessions (p<0.05) as compared to the control group. | | Results are not generalizable to all adolescent groups e.g. adolescent boys, adolescents living in rural areas and non-school going. |
| C [44] | Uttar Pradesh | | | 2002 | | | CARE India (India chapter of international NGO) | | | Foster the development of alternative socialization processes for adolescent girls that enhance the development of positive sexual and reproductive health behaviors  Integrate vocational counseling, training, and follow-up support for adolescent girls coupled with encouragement of savings formation into CARE’s Action for Slum Dwellers’ Reproductive Health project in Allahabad  Increase participation by adolescent girls in other reproductive health-related activities of the Action for Slum Dwellers’ Reproductive Health, Allahabad (ASRHA) Project (e.g., sexual health, hygiene, and nutrition).  4. Foster community acceptance of physical mobility by adolescent girls, strengthen and enlarge positive peer-to-peer support networks, and develop new mentor relationships between younger and older women. | Quasi-experimental: pre- and post-test design comparing intervention and control group | | | **Inputs**  *Intervention*: Multi-component package offering livelihoods, savings formation, reproductive health and communication skills  *Target population*: Adolescent girls between the ages of 14 and 19 years residing in urban slums of Allahabad  *Human resources*: Study team, consultants, AGGs (adolescent girl guides/peer educators) and AGG assistants  **Process**  The reproductive health training sessions were conducted using a set of five specially designed flipbooks in Hindi that related to the experiences of a 12-year-old girl. The flipbooks covered the following topics: physiological and behavioral changes during puberty; menstruation; pregnancy and birth; age at marriage and birth spacing; and family planning, including husbands’ roles.  The AGGs were responsible for forming groups of peers from their communities and for conducting reproductive health sessions, which were held at their own residences. Each flipbook was completed in 1-2 hours, depending on the girls’ participation. In addition to reproductive health information, AGGs provided counseling regarding vocational training and savings formation.  The study team was present at the meetings to help the AGGs educate the girls. In addition, eight assistant peer educators were trained to support the AGGs during the intensive group work. The effectiveness of the intervention was assessed with a follow-up survey conducted 8 months after the baseline survey. | | **Outputs**  *Coverage*: Information on coverage was not mentioned    *Quality assessment:* No results of quality assessment were reported.  **Outcomes**  Increase in adolescent girls’ knowledge of reproductive health between baseline and follow-up surveys, including:   - being able to correctly name one or more contraceptive methods (89% to 97%), - being able to name an STI (67% to 94%), - being able to correctly answer a question about duration of pregnancy (76% to 100%), and knowing sexual contact was required to become pregnant (44% to 98%). | | High lost to follow-up rate; 62 cases matched between baseline and midline |
| D [45] | Uttar Pradesh (UP) and Delhi National Capital Region (NCR) | | | 2015 | | | The YP Foundation (Indigenous NGO) | | | Scale 1760 young people’s knowledge of and access to Sexual and Reproductive Health and Services and Life Skills by implementing programs on the same through a peer-to-peer approach at community levels with both in and out-of-school young people.  Strengthen the demand for Sexual and Reproductive Health Services at village, block and district levels, using existing service delivery platforms through community sensitization for adolescent and youth friendly preventive, promotive and curative services.  Advocate for implementation of peer education both in school and out of school at the state level in Uttar Pradesh and advance National Level dialogue and policy advocacy with key civil society actors, UN agencies and government ministries. | Descriptive | | | **Inputs**  *Intervention*: Comprehensive sexuality education (CSE) and community outreaches  *Target population*: Young people across two states in 14 villages across Jhansi and Lucknow districts in UP and 12 low-income sites in Delhi NCR.  *Human resources*: Trained peer educator and YP staff members  **Process**  The CSE curriculum covered a range of topics including gender and sex; anatomy; puberty, menstruation; masturbation; relationships, negotiation and consent; violence; sexuality; conception, contraception and abortion; and sexually transmitted infections, including HIV. Its development built on existing best practices for those aged 10 to 14 and 15 to 18. The sessions were designed to increase participants’ knowledge, to encourage critical thinking about gender, to affirm body-positive attitudes and to learn how to exercise their rights.  The curriculum was 25 hours in total and was imparted by peer educators during 2-hour long, weekly workshops to young people. A total of 400 workshops were conducted by peer educators both in-school and out-of-school settings in partnership with 22 civil society organization (CSOs).  In addition to CSE, peer educators and partnering CSOs planned their strategies for outreach to marginalized communities to create demand. In Jhansi, the intervention included puppet shows, street theater and a district-level dialogue and rally. In Lucknow, street theater, district-level dialogue with stakeholders and 1-to-1 meetings with service providers were utilized. In Delhi NCR, the intervention included role-plays, poster making, film screenings, engagement of participants with peers on specific issues such as HIV/AIDS, healthy relationships and domestic violence. | | | **Outputs**  *Coverage*: CSE workshops were conducted with 914 participants in Delhi NCR and 1,154 participants across Jhansi and Lucknow. No information on denominators was mentioned.    *Quality assessment:* No results of quality assessment were reported.  **Outcomes**  Increased knowledge on sexual reproductive health information and services amongst 1750 young people in the NCR, Lucknow and Jhansi – no metrics were provided in the report.  Increase in community sensitization for adolescent and youth friendly preventive, promotive and curative services in Delhi NCR, Lucknow and Jhansi.  Increase in advocacy for implementation of peer education both in school and out of school. | Accurate change in participant knowledge difficult to capture due to young participants’ unfamiliarity with the issues in survey questions |
| E [46] | West Bengal | | | 2015 | | | CINI (Indigenous NGO) | | | *Out of School: Link Worker Scheme*  Increase knowledge and skills on SRH including gender, HIV and AIDS, RTI/STI, correct contraceptive usage and safe abortion services among young people  Improve knowledge on Pre-Conception and Pre-Natal Diagnostic Techniques Act (PCPNDT Act, 1994).  Increase in consistent and correct condom use among young people to reduce STI and HIV transmission  Contribute to reduced unplanned pregnancies and unsafe abortions    *In School: Health Program*  Build capacity of government school teachers on Life Skill Education (LSE) including SRH and nutrition  Increase knowledge, life skills and awareness on SRH and nutrition rights and services (including related to HIV and AIDS and gender) for adolescent girls  Provide technical support to Government of West Bengal for integrating LSE in school-based curriculum linked to right to education, leading to sustainable and scalable framework | Quasi-experimental: pre- and post-test design | | | **Inputs**  *Intervention*: Multicomponent intervention to advance young people’s sexual and reproductive health and rights through government - civil society partnership  *Target population*: Adolescents in school and out of school in 4 districts of West Bengal  *Human resources*: Link workers, CINI team members, block and district level health functionaries including Anwesha counsellors (lady health counselors at adolescent health clinics), school teachers and peer educators  **Process**  The topics covered in both out of school and in school interventions included: adolescence; puberty; nutrition; and key SRH issues such as reproduction, contraception, safe abortion, reproductive tract and sexually transmitted infections and HIV.  Trained peer educators were involved in disseminating this information through both the in-school and out-of-school components of the programme and making linkages with services for young people. The out-of-school link workers scheme had a focus on reaching out to vulnerable adolescents in rural areas through peer educators, whilst the School Health Programme focused on building the capacities of school teachers to delivery Life Skills Education (LSE) in addition to peer education.  CINI’s interventions also included project sensitization meetings with key stakeholders such as functionaries of the Ministry of Health and Family Welfare (MoHFW), Integrated Child Development Services (ICDS) and the Education Department. Health system functionaries such as counselors, block-level medical officers, auxiliary nurse midwives and accredited social health activists (ASHAs) were also included in such meetings to in order to enhance linkages with services. Additionally, the project capitalized on events (e.g. World AIDS Day), health fairs and Village Health and Nutrition Days to galvanize local communities.  The effectiveness of CINI’s intervention was assessed through an endline survey conducted 3 years after the baseline survey. | | **Outputs**  *Coverage:*   - Out of school: Peer educators reached out to 10,000 adolescents and young people across 200 villages in 2 districts; no information on the denominator was provided - In school: Peer educators reached out to 3,824 students directly who, in turn, reached out to another 123,407 indirectly across the 2 districts; no information on the denominator was provided   *Quality assessment*: During the implementation CINI team members conducted process documentation, but no results were included in the report. No results of quality assessment were reported.  **Outcomes**  *Out of School:* Increases in knowledge were reported for –   - Contraceptives as protection against pregnancy (Burdwan district: 34% to 87%; North Dinajpur: 22% to 76%) - Prevention of HIV & AIDS (Burdwan district: 73% to 78%; North Dinajpur: 49% to 87%) - HIV testing (Burdwan district: 29% to 83%; North Dinajpur: 20% to 91%) - Legal age at marriage for girls (Burdwan district: 29% to 92.5%; North Dinajpur: 23% to 94.5%) - Legal age at marriage for boys (Burdwan district: 26% to 77%; North Dinajpur: 52% to 86%)     *In School:* Increase in knowledge was reported for –   - Contraceptives as protection against pregnancy (South 24 Paraganas: 32% to 71.5%; Murshidabad: 22% to 72.5%) - Prevention of HIV & AIDS (South 24 Paraganas: 55% to 73%; Murshidabad: 80% to 82%) - HIV testing (South 24 Paraganas: 50% to 78.5%; Murshidabad: 75% to 87%) - Legal age at marriage for girls (South 24 Paraganas: 29% to 95%; Murshidabad: 30% to 96.5%) - Legal age at marriage for boys (South 24 Paraganas: 60% to 94.5%; Murshidabad: 71% to 94.5%) | | *Out of School*  Deep rooted social norms and taboos related to discussion on SRH issues and participation of girls in activities took time to transcend  Link workers working at fixed remuneration for many years, affecting motivation    *In School*  Hesitation among school authorities and teachers in dealing with SRH content particularly in co-ed schools  Lack of sufficient teachers in schools impacting workload of Master trainers; also their varying backgrounds and interest levels  High dependence on CINI to implement the peer education. |
| F [47] | West Bengal | | 2016 | | | CINI (Indigenous NGO) | | To strengthen the implementation of the Adolescent Health Strategy [RKSK] of Government of India to contribute to the overall development of the adolescents in the state of West Bengal  To build capacity of adolescent girls and boys on reproductive sexual health and rights, hygiene, nutrition, non-communicable diseases, substance misuse, psychosocial health, gender-based violence free lifestyle through strengthening convergence between different Government adolescent development programs of different Departments like Health and Family Welfare, Women and Child Development and Education  To empower adolescent girls and boys on technical aspects like identifying vulnerability issues, planning, and leadership so that they can participate in local convergence platforms  To enhance technical capacity of service providers and local self-government to lead a sustainable process of adolescent development in their area through convergence between different programs for adolescents  Evidence-based documentation of the holistic, scalable and sustainable model of a common adolescent development program to be replicated at state and national levels through Government-Civil Society partnership | | | | | Descriptive | **Inputs**  *Intervention*: Sensitization on the six RKSK components  *Target population*: Adolescent girls and boys aged 10 to 19 years living in four community development blocks of two resource poor districts in West Bengal: South Twenty-four Parganas and Murshidabad in the State of West Bengal  *Human resources*: CINI staff members, peer educators, peer educator master trainers from local NGOS  **Process**  The six components of RKSK are: nutrition, sexual and reproductive health (SRH), non-communicable diseases (NCDs), substance misuse, injuries and violence (including gender-based violence) and mental health. Over the period of a year, peer leaders conducted interactive sessions, trainings and role plays as well as shared information, education and communication materials to impart knowledge about SRH adolescents in their respective localities.  Each peer leader was assigned 10 adolescents and were given tracking sheets to monitor ‘risky’ behaviour, health practices and problems. A mix of qualitative (focus group discussions, in-depth interviews) and quantitative research (close-ended questionnaire) was used for impact assessment. The impact assessment was conducted 10 months after the implementation of the intervention. | | **Outputs**  *Coverage*: 136,153 (male = 61,507; female= 74,646) adolescents were sensitized about the RKSK components, from a desired aim of reaching 180,000 adolescents    *Quality assessment*: The sustainability of the project was ensured by monitoring the activities of peer leaders regularly. A community monitoring mechanism was also developed that involved administrative officials, health officials, parents, teachers and adolescents and together they keep a vigil on adolescents in the locality. No results of quality assessment were reported.  **Outcomes**   - More than 5,585 cases referred to the Adolescent Friendly Health Clinics by peer leaders and master trainers to access counseling and condoms - Improved SRH awareness and practices among adolescents - Improvement in SRH awareness and practices, namely related to onset of menstruation; menstrual hygiene; prevention SRH related ailments; maintaining personal hygiene and eating nutritious food; and significant increase in in use of sanitary napkins - Increased reporting of SRH-related issues by adolescents, including higher reporting of menstrual problems from adolescent girls | | Need for support in terms of refresher trainings on adolescent topics as there was a general feeling of “not knowing enough”  Need for aids to conduct sessions – video, IEC and other interactive materials so as to make the knowledge transfer better and uniform  Insufficient training for peer educators.  Lack of resources for standardized high-quality knowledge transfer. |
| G [48] | Uttar Pradesh (UP) | | | 2003 | | | Population Council (International NGO) and CARE India (India chapter of international NGO) | | | To examine whether an experimental intervention for girls aged 14–19 that provided vocational counseling and training and assistance with opening savings accounts in slum areas of Allahabad  Increased physical mobility and contact with individuals outside the family as well as awareness of safe places for girls to congregate  Increased self-efficacy  Increased reproductive health knowledge  Altered work aspiration and encouraged more progressive gender role norms  Reduced time spent on domestic tasks and increased time spent on productive tasks | Quasi-experimental: pre- and post-test design comparing intervention and control groups | | | **Inputs**  *Intervention*: Multicomponent intervention including provision of reproductive health information, vocational counseling and training, and assistance with opening savings accounts  *Target population*: Adolescent girls 11 to 18 years of age residing in the urban slums of Allahabad  *Human resource*s: Staff members of CARE, Population Council, and the Centre for Operations Research and Training (CORT), as well as peer educators  **Process**  The intervention, which began in 2001, integrated livelihoods activities for adolescent girls aged 14 to 19 years into CARE’s reproductive health program for slum dwellers known as ‘Action for Slum Dwellers’ Reproductive Health, Allahabad’ (ASRHA). The reproductive health curriculum used included the following topics: puberty, menstruation, reproductive biology, pregnancy, contraception, sexually transmitted infections and age at marriage.  Each trained peer educator was expected to visit every household in her locality and invite all eligible girls (11 - 18 years old) to participate in the project. When approximately 20 girls were ready to participate, a group was formed and met at the home of a peer educator. Participants residing in the experimental slums received the reproductive health training sessions and follow-up support from a peer educator. The vocational counseling and savings components were provided by project staff after the completion of the reproductive health curriculum and were open only to those participants who had maintained good attendance and were in the 14 to 19 years age bracket.  CORT conducted the census of the selected slums and data collection for baseline and endline surveys. The duration between the baseline and endline survey was 3 years. | | **Outputs**  *Coverage*: Peer educators reached 525 adolescent girls out of 1,683 eligible adolescent girls interviewed at baseline    *Quality assessment*: No results of quality assessment were reported.  **Outcomes**  There were significant improvements in the following:   - Increase in knowledge of safe spaces for girls: intervention 83.2% vs. control matched 33.7% (p ≤0.001) - Increase in reproductive health knowledge (mean score): intervention 6.7 vs. control matched 5.7 (p ≤0.001) - Increased social skills (mean) and group membership: intervention 12.0 vs control matched 11.0 (p ≤0.05) and intervention 15.6% vs. control matched 5.1% (p ≤0.01) respectively - Increased time spent on leisure activities (mean): intervention 4.4 vs control matched 3.7 (p ≤0.05)     There were no significant effects found on gender-role attitudes, mobility, self-esteem, work expectations, or on number of hours visiting friends, performing domestic chores, or engaging in labor-market work. | | Difficult to conduct a longitudinal survey in an urban slum area – unable to match participants from baseline to endline  Intervention duration and intensity insufficient to produce sizeable effect  Outcome variables used to evaluate the impact of the intervention were not appropriate |
| H [49] | Two districts of Jharkhand – Gumla and Ranchi | | | 2013 | | | The Centre for Development and Population Activities (CEDPA) (Indigenous NGO) | | | To examine CEDPA India’s role in the provision of technical assistance at the state level and experiences in implementation of the SABLA scheme in Gumla as a case study and recommend a framework for scaling up of the scheme at the national level  To assess change among select adolescent girls at the personal level  To assess innovations brought in by CEDPA India for effective implementation of the scheme and  adequacy, efficiency and effectiveness of the innovations  To assess gaps and challenges in effective implementation of SABLA  To recommend framework for scale up | | Quasi-experimental: pre- and post-test design comparing intervention and control group | | | **Inputs**  *Intervention*: Implementation of SABLA scheme with a focus on nutrition and sexual and reproductive health (SRH)  *Target population*: Adolescent girls aged 11 to 18 years  *Human resources*: Staff from CEDPA India, child development project officers at the district level, lady supervisors, local NGOs, and peer educators (sakhis [peer monitor]/sahelis [peer leaders])  **Process**  A series of activities were undertaken, including a baseline survey, to assess the current context of adolescent girls; the training and capacity building of Anganwadi Workers (AWWs), Sakhi and Sahelis; and monitoring of vocational training for the adolescent girls participating in the Scheme. SABLA helped implement the scheme in two districts of Ranchi and Gumla, with a control group in Bokaro.  CEDPA undertook a training at the district level of child development project officers, lady supervisors, and NGOs from both districts. These workers then trained adolescent girls who implemented the scheme at the village level. CEDPA facilitated another training of Sakhis/Sahelis to inform them about SABLA Scheme and train them to conduct sessions in their respective groups (Samuhs). CEDPA developed a ready reckoner for Sakhis/Sahelis, which they and anganwadi workers used regularly to update their knowledge before a Kishori Samuh meeting.  The duration between the baseline and follow-up evaluation was approximately 12 months. | **Outputs**  *Coverage*: Not mentioned    *Quality assessment:* CEDPA regularly monitored and provided technical assistance to Sakhi, Sahelis, and Anganwadi workers. They also liaised with the child development project officers and the supervisors on a regular basis*.* No results of quality assessment were reported.  [All results are not available as this was a midline report, with only qualitative findings emerging out of it. There was no mention of the impact of the SABLA scheme in the control group]  **Outcomes**   - Both AWWs and peer educators appreciated the way the training was facilitated and they enjoyed the activities, role plays and games played during the training; they found the program useful and easy to understand - Sakhis and Sahelis who underwent the three days’ training reported that it was informative and useful - The facilitators and the girls felt that even though the programme is new, participating in SABLA Scheme was a positive experience - There was an increase in knowledge and confidence levels amongst the girls, who could negotiate and solve problems and communicate more effectively with their parents - Adolescent girls became more aware about various issues like nutrition, menstrual hygiene and reproductive health | | No clear-cut guidelines or uniformity in the organization of Kishori Samuh meetings  AWWs find it difficult to dedicate time to Kishori Samuhs due to the multitude of responsibilities entrusted to them under the ICDS programme  Permissions needed for implementation from relevant government officials on an ongoing basis; the process was time consuming |
| I  [50] | Bihar | 2015 | | | Centre for Catalyzing Change (indigenous NGO), Population Council (International NGO), and London School of Hygiene & Tropical Medicine (Academic institution) | | | | | 1) To measure the impact of the Do Kadam Barabari Ki Ore (Two Steps Towards Equality) project among boys in ages 13–21 years, who were members of youth clubs supported by the NYKS program of the Ministry of Youth Affairs and Sports, in:  Changing gender role attitudes, attitudes toward violence against women and girls, and controlling behaviors over sisters, girlfriends, and wives  Reducing the perpetration of various forms of violence against women and girls  2) To assess the feasibility and acceptability of the intervention | Randomized control trial with in-depth interviews and panel surveys | | | **Inputs**  *Intervention*: Multi-component intervention comprised of gender transformative life skills education combined with cricket-coaching  *Target population*: Adolescent boys aged 13 to 21 years  *Human resources*: Centre for Catalyzing Change (C3) staff members and peer mentors  **Process**  Drawing on previous, successful programmes for boys, the Do Kadam intervention focused on promoting egalitarian gender attitudes and abhorrence of violence against women and girls through gender transformative life skills education combined with cricket-coaching.  The intervention was delivered over a period of 18 months through 42 weekly sessions, discounting festivals and holidays. One hour each week was devoted to gender transformative life skills education, which was delivered by peer mentors, and one hour to cricket-coaching delivered by C3 staff members. Peer mentors used the training module to deliver each session, using the guidelines and participatory methodologies recommended for each session. C3 core trainers supported peer mentors to ensure that sessions were conducted as per the guidelines, that questions raised by participants were answered adequately, and that peer mentors were able to maintain discipline during the sessions.  Participants were surveyed both at baseline and at endline. The duration between the two surveys was 2 years. | | **Outputs**  Coverage: Peer mentors reached out to 516 boys in 15 youth clubs in the intervention group out of 583 eligible unmarried boys interviewed at baseline    *Quality assessment*: No results of quality assessment were reported.  **Outcomes**  Boys in the intervention arm expressed egalitarian gender role attitudes and notions of masculinity in 6.6 of 9 attitudes probed, compared to 6.2 reported by those in the control arm (effect estimate 0.40, p ≤0.05)  Boys in the intervention arm rejected men’s and boys’ right to exercise control over women in 4.9 situations, compared to 4.2 situations reported by those in the control arm (effect estimate 0.7, p ≤0.01)  Of 17 situations probed, boys from the intervention arm rejected the right of men and boys to exercise violence against women and girls in 11.7, compared to 10.3 among those in the control arm (effect estimate 1.3, p ≤0.001)  In terms of perpetration of various forms of gender-based violence, there was weak evidence that non-contact forms of violence, such as stalking a girl, had declined because of the intervention. However, in in-depth interviews, several boys’ narratives suggested at the endline interview that they no longer participated in teasing girls and some of them specifically attributed the change they had experienced directly to what was conveyed in the Do Kadam programme. | | Number of sessions too few to identify change  Peer mentors lacked confidence and communication skills, and were uncomfortable conveying  sensitive messages such as those related to sexual violence |
| J  [51] | Maharashtra | | | 2008 | | | Albert Einstein College of Medicine, Yeshiva University, New York and Drug Abuse Information, Rehabilitation and Research Center, Mumbai (Academic Institution and Indigenous NGO) | | | To test whether an educational program built on specific cultural, linguistic, and community-specific characteristics was effective in improving knowledge, beliefs, and attitudes about HIV infection in general and increased confidence in youth (8 grade students, 13-21 year old) in dealing with high-risk situations. | Quasi-experimental: pre- and post-test design comparing intervention and control group | | | **Inputs**  *Intervention:* School-based Teenage Education Program (STEP) focusing on HIV Prevention  *Target population:* Eighth grade students (13-15 years old) in 25 schools randomly selected in Mumbai  *Human resources*: Peer educators, staff members from implementing organization (DAIRRC)  **Process**  The educational programme was developed by combining curriculum from successful HIV prevention programs in the United States with existing curriculum-based drug education efforts modeled on the social learning theory of Drug Abuse Information, Rehabilitation and Research Center (DAIRRC). The programme was developed to improve knowledge, beliefs and attitudes about HIV infection and increase young people’s confidence in dealing with high-risk situations.  Trained peer educators conducted the STEP program over 6 weeks (single one-hour session per week for six consecutive weeks) in participating schools. Two classes in each school participated, and the school administrator randomly assigned the classes to intervention and control arms. Students in the intervention group were exposed to the STEP curriculum, while those in the control arm received no curriculum.  The baseline survey was completed by 1,846 students (946 intervention and 900 control), and the endline survey was completed by 1,733 (882 intervention and 859 control). The duration between the baseline survey and endline survey was 6 weeks. | | **Outputs**  *Coverage*: 1800 students in 25 Mumbai schools    *Quality assessment*: No results of quality assessment were reported.  **Outcomes**  Knowledge of HIV/AIDS: Students who received STEP curriculum evidenced greater knowledge after 6 sessions (p≤.001); girls had lower knowledge at baseline but significantly higher compared to boys at the end of intervention (p<.05)  Beliefs: change in belief towards more tolerance, more pronounced in the intervention group (p≤.001; both genders reported improved positive beliefs towards people living with HIV/AIDS (p<.001)  Attitudes: difference in attitudes significant in the intervention group for four attitudes - abstinence (p<.01), using condom consistently (p<.001), understanding of precautions (p<.05), less likely to be influenced by peers (p<.001)  Confidence: assessed refusal skills in dealing with peer and social pressure. Differences more significant and pronounced for intervention group (p≤.001) | | Most trainers in the program were females  Intervention and control groups were in the same school  Findings are not generalizable to rural area, as intervention took place in metropolitan area |
| K [52] | Maharashtra and Uttar Pradesh | | | 2008 | | | Committee of Resource Organizations (CORO) for Literacy, MAMTA and DAUD (International and Indigenous NGOs) | | | To reduce HIV vulnerability among men and women and to reduce young men’s use of violence against women and girls through the promotion of gender equitable attitudes and behaviors  To adapt the Gender Equitable Men (GEM) Scale, initially tested and developed in Brazil, to the Indian context for use as an evaluation tool.  To test the impact of peer-led group educational activities and community-based social marketing campaigns in promoting gender equitable attitudes and behaviors and safe sexual practices among young men from low-income communities in Mumbai.  Adapt and test the effectiveness of peer-based group educational activities among young men in rural settings in Gorakhpur, Uttar Pradesh. | Quasi-experimental: pre- and post-test design comparing intervention and control group | | | **Inputs**  *Intervention*: Group education sessions (GES) and lifestyle social marketing campaign (LSSM) delivered through peer education, collectively referred to as ‘Yaari-Dosti.’  *Target population*: married and unmarried men age 15-29 in urban and rural settings from Mumbai, Maharashtra and Gorakhpur, Uttar Pradesh respectively.  *(Note: There is no information on the proportion of 24-29 year olds. We included the study as it was a well written report showcasing knowledge, attitudinal and behavioral outcomes.)*  *Human resources*: CORO for Literacy and MAMTA staff members in Mumbai and Gorakhpur and peer educators.  **Process**  The themes covered through the GES were: gender and sexuality: STIs and HIV risk and prevention; violence; reproduction; alcohol and risk; and HIV-related stigma and discrimination. The topics were covered using participatory learning methods, including games and role-playing, that engaged the participants in discussion, debate and critical thinking. The GES were implemented over 6 months (one session per week) by peer educators, each session lasting for over one hour. Facilitators and field supervisors, together with gender experts, met once every month to discuss and share experiences about implementation.  The LSSM component reinforced messages from the GES and focused on gender-equitable lifestyles and versions of manhood through community-based activities conducted in spaces where young men congregate. This was delivered through street plays; posters; and distribution of pamphlets, comic strips, community-based discussions, t-shirts and condoms at a ‘mobile booth.’ The LSSM campaign was developed and led by peer educators using information gathered during the formative research phase and subsequent testing of messages in the community. The campaign developed materials based on young men from these communities that projected an alternative form of masculinity.  The effectiveness of the intervention was assessed through a survey at baseline and follow-up survey approximately 6 months later. | | **Outputs**  *Coverage*: Peer-educators reached out to 1,178 peers out of 1,195 young men in the chosen communities    *Quality assessment:* No results of quality assessment were reported.  **Outcomes**  A total of 1,195 (875 Mumbai + 1040 Gorakhpur) completed the baseline survey and 1,138 (537 Mumbai + 601 Gorakhpur) completed the follow-up survey. Some of the notable outcomes were:  Communication with partners on condoms, sex, STIs, and/or HIV significantly improved in the intervention sites.  Improvements among the intervention participants in discussing key reproductive and sexual health issues (condom use, sexual relationships, STIs, and HIV/AIDS) with a female partner in the last three months (p <0.05)  There was a significant increase in condom use at last sex with all partner types in the intervention areas (p <0.05)  Men in the intervention arms were 1.9 times more likely to have used condoms at last sex in Mumbai (p <0.001) and 2.8 times more likely to have used them in Gorakhpur (p <0.001) than those in the comparison arms in each setting  Report of sexual health problems during the previous three months decreased significantly in the intervention sites from baseline to follow-up (p <0.05)  There was a positive trend toward improvements in GEM Scale scores being associated with decreases in HIV/STI risk behaviors (not significant)  Young men in the intervention sites reported more positive attitudes toward person living with HIV (p <0.05) | | Self-selection of the study participants  Outcome measures relied on self-reports of participants |
| L [53] | 12 states: Uttar Pradesh, Haryana, Punjab, Bihar, Odisha, Maharashtra, Rajasthan, Karnataka, Andhra Pradesh, Tamil Nadu, Assam, Tripura | | | 2013 | | | Administrative Staff College of India (Academic institute) | | | To evaluate the Rajiv Gandhi Scheme Empowerment Adolescent Girls - Sabla  To assess the scheme from the perspective of functioning of the scheme and all its components including nutrition component; non-nutrition component; administrative  component; flow of funds; training component; publicity component; adolescent girls being serviced through the scheme; role of government officials (State level, district level, block level and village-level) their response towards the scheme; non-beneficiaries; and other stakeholders including families of the AGs and PRI members, Community leaders of the village  To assess the strengths of the scheme and barriers against the scheme  To provide a ‘way forward’ for the scheme | Descriptive | | | **Inputs**  *Intervention*: Multi-component scheme comprised of nutrition and non-nutrition educational sessions, life-skills sessions and vocational training  *Target population*: Adolescent girls 11 to 18 years of age  *Human resources*: Anganwadi workers (AWW), AWHs, department of health officials, NGOs, self-help groups, Child Development Project Officer, adolescent girl peer monitors and educators  **Process**  This multi-component intervention focused on improving the health and nutrition status of adolescent girls by raising their awareness about health, hygiene, nutrition, adolescent reproductive and sexual health (ARSH), family and childcare. Adolescent girls also received life skills education and vocational training.  The nutrition component of the scheme was delivered by AWWs, who also supported the peer monitors (sakhis) and peer leaders (sahelis) to deliver the life skills and vocational trainings in places where adolescent girls receive information. Adolescent girls also made social connections with their peers; built confidence and morale; and received support for envisioning their futures through the scheme.  The evaluation relied on a mix of quantitative and qualitative methods. It was designed to be conducted in 12 states distributed across the five regions (at least 2 from each) of the country over a two-year period. The selection of states was based on the pilot districts and states where the scheme had been implemented. The fieldwork phase included field survey with beneficiaries conducted largely at anganwadis or in a school hall; focus group discussions with beneficiaries and with community members; and key informant interviews with officials, staff and others. | | **Outputs**  *Coverage*: coverage of nutrition component was 1.13 crores beneficiaries out of a target of 10,170,443; coverage of family welfare, SRH and childcare practices increased from 3,592,506 adolescent girls in 2011-12 to 4,811,264 adolescent girls in 2012-13    *Quality assessment:* In order to ensure  effective implementation and monitoring of the Sabla throughout the country, a National  Monitoring and Supervision Committee has been set up under the chairpersonship of the Secretary, Ministry of Women & Child Development. This Committee meets quarterly or as and when required at the notice of the Chairperson. No results of quality assessment were reported.  **Outcomes**  71.6% of the total respondents had ever attended ARSH counselling out of 3,358 out of school girls surveyed  Out of out of 2,322 adolescent girls surveyed:   - 96.4% AGs reported that the counselling sessions were useful or very useful - 70.9% of the AGs reported having some knowledge about HIV/AIDS - 87% claimed that they would visit a doctor when pregnant. - 75.2% of respondents were aware of Kishori Samooh   Around 85.2% sakhis and sahelis (peer mentors) reported a sense of ownership of the delivery of activities under the Sabla scheme out of 424 and 780 sakhis and sahelis surveyed. | | In the absence of baseline, and given the short span since program implementation, and the short study timeline, the impact questions from the beneficiary perspective could not be fully investigated such as nutritional impacts or behavioral and change in practices.  Ineffectiveness of the anganwadi workers in motivating the adolescent girls to attend the anganwadi regularly.  Limited infrastructure in AWWs to organize Kishori meetings  Improper reporting of scheme components and difficulty in filling forms and consolidating data was observed  Low involvement of sakhis and sahelis in the scheme |
| M [54] | Jharkhand | | | 2015 | | | Centre for Catalyzing Change (C3) (Indigenous NGO) | | | To increase the acceptability of and access to family planning methods by married adolescent couples by providing knowledge and information on modern contraceptive methods to enable them to plan and limit their family size, in Ramgarh district of Jharkhand, India    To ensure effective linkages between eligible couples and service providers for accessing Reproductive Health Services including Family planning services | Quasi-experimental: pre- and post-test design | | | **Inputs**  *Intervention*: Information on reproductive health practices such as family planning and accessing reproductive health services  *Target population*: Married adolescent couples – girls (10-19 years) and their husbands (14-25 years) residing in Ramgarh district  *Human resources*: Project personnel from C3 team and Nav Bharat Jagrity Kendra, 50 trained peer educators  **Process**  The intervention was designed to increase knowledge and awareness of age of marriage, reproductive health practices, delaying first pregnancy, use of family planning methods, linkages with service providers, and access and availability of reproductive health services in the health facilities. A total of 3,038 meetings (2 meetings/fortnight/village/month) were conducted during the period with married adolescent couples in 50 target villages. The peer educators gave the scheduled sessions among the married adolescent couples with support from health facilitators, who are functionaries of the Department of Health and Department of Women and Child Development. The group meetings were organized and held separately for the wives and the husbands due to their availability.  The effectiveness of the intervention was assessed through a survey at baseline and an endline survey 12 months later. | | **Outputs**  *Coverage*: 950 married adolescent couples were identified and reached from approximately 50 villages (25 in each block – Ramgarh Sadar and Patratu); no information on denominators available    *Quality assessment:* No results of quality assessment were reported.  **Outcomes**  *Knowledge:*  Increase in awareness among married adolescent girls (MAGs) e.g. for: contraceptive pills (Baseline: 20%, Endline: 91%), condoms (Baseline: 26%, Endline: 91%), injectable contraceptives (Baseline: 10%, Endline: 36%)  Increase in awareness among husbands on married adolescent girls (HMAGs) e.g. for: condoms (Baseline: 39%, Endline: 97%), vasectomy (Baseline: 15%, Endline: 56%), tubectomy (Baseline: 16%, Endline: 59%), contraceptive pills (Baseline: 31%, Endline: 69%), Emergency Contraceptive Pills (Baseline: 8%, Endline: 38%)  Increase in awareness of pregnancy detection kit (Baseline: 28%, Endline: 86%) and awareness of its use as well (Baseline: 20%, Endline: 56%)  Increase in awareness of complication in pregnancy and childbirth often leading to maternal death as a consequence of early pregnancy: among MAGs (Baseline: 67%, Endline: 87%) and HMAGs (Baseline: 74%, Endline: 96%)  Improved knowledge of AFHC clinic both among MAGs and HMAGs  Increase in awareness of benefits provided by Government in case of institutional delivery amongst:   - MAGs: free transportation to and from the health facility (Baseline: 21%, Endline: 100%), free stay at the health facility (Baseline: 23%, Endline: 100%), free medicines (Baseline: 30%, Endline: 100%), cash benefit (Baseline: 18%, Endline: 100%) - HMAGs: free transportation to and from the health facility (Baseline: 36%, Endline: 97%), free stay at the health facility (Baseline: 38%, Endline: 99%), free medicines (Baseline: 47%, Endline: 99%), cash benefit (Baseline: 34%, Endline: 98%)   Increase in awareness for ANC, delivery and PNC services for women amongst:   - MAGs: dietary supplement (Baseline: 66%, Endline: 100%), TT immunization (Baseline: 67%, Endline: 100%), iron folic acid tablets (Baseline: 68%, Endline: 100%), ANC checkup (Baseline: 68%, Endline: 100%), delivery (Baseline: 64%, Endline: 100%), PNC (Baseline: 64%, Endline: 99%) - HMAGs: dietary supplement (Baseline: 89%, Endline: 99%), TT immunization (Baseline: 89%, Endline: 99%), iron folic acid tablets (Baseline: 85%, Endline: 98%), ANC checkup (Baseline: 85%, Endline: 99%), delivery (Baseline: 86%, Endline: 99%), PNC (Baseline: 76%, Endline: 96%)   *Attitude:*  Increase in preference to visit a health facility for symptoms like vaginal discharge, burning micturition, genital itching, genital ulceration and pain in lower abdomen among MAGs (Baseline: 46%, Endline: 93%)  Increase in preference to visit a health facility for symptoms like vaginal discharge, burning micturition, genital itching, genital ulceration and pain in lower abdomen among HMAGs (Baseline: 51%, Endline: 92%)  *Behavior:*  MAGs reported increase in being advised by ASHA/ANM on delaying first pregnancy (Baseline: 8%, Endline: 55%)  HMAGs reported being advised by ASHA/ANM to delay the first pregnancy (Baseline: 2%, Endline: 18%)  Increase in using a contraception method (Baseline: 14%, Endline: 59%)  Increase of MAGs going to the first point of contact, the local ANM, for confirmation of pregnancy (Baseline 14%, Endline: 75%)  Increase in AFHS visits (Baseline: 0% vs Endline: 50%) | | Loss to follow-up of MAGs between baseline and endline surveys |
